# Exploring the green development path of the Yangtze River Economic Belt using the entropy weight method and fuzzy-set qualitative comparative analysis

**DOI:** 10.1371/journal.pone.0260985

**Published:** 2021-12-06

**Authors:** Haijuan Yan, Xiaofei Hu, Dawei Wu, Jianing Zhang

**Affiliations:** 1 School of Management, Nanchang University, Nanchang, China; 2 School of Economics and Management, Nanjing University of Science and Technology, Nanjing, China; University of Defence in Belgrade, SERBIA

## Abstract

Green development is an effective way to achieve economic growth and social development in a harmonious, sustainable, and efficient manner. Although the Yangtze River Economic Belt (YREB) plays an important strategic role in China, our understanding of its spatiotemporal characteristics, as well as the multiple factors affecting its green development level (GDL), remains limited. This study used the entropy weight method (EWM) to analyze the temporal evolution and spatial differentiation characteristics of the GDL in the YREB from 2011 to 2019. Further, fuzzy-set qualitative comparative analysis (fsQCA) was used to analyze the influence path of GDL. The results showed that the GDL of the YREB increased from 2015 to 2019, but the overall level was still not high, with high GDL mainly concentrated in the lower reaches. The GDL model changed from being environmentally driven and government supported in 2011 to being environmentally and economically driven since 2014. The core conditions for high GDL changed from economic development level (EDL) to scientific technological innovation level (STIL) and environmental regulation (ER). The path for improving GDL is as follows: In regions with high EDL, effective ER, moderate openness level (OL), and high STIL are the basis, supplemented by a reasonable urbanization scale (US). In areas with low EDL, reasonable industrial structure (IS) and STIL are the core conditions for development; further, EDL should be improved and effective ER and OL implemented. Alternatively, without considering changes to EDL, improvement can be achieved through reasonable OL and US or effective ER. This study provides a new method for exploring the path of GDL and a reference for governments to effectively adjust green development policies.

## 1 Introduction

Rapid economic growth has caused extensive environmental harm throughout the world. To resolve the contradiction between ecological conservation and economic development, the United Nations proposed the concept of sustainable development in 1987, calling on all countries to pursue development without jeopardizing the living needs of future generations [1]. Green development is a theoretical innovation of sustainable development, which balances and coordinates local economic growth, social development and ecological environment improvement [2]. The green development concept has gained wide currency globally since 2008 [1–7]. Many countries, including the Canada, United Kingdom, Germany and South Korea have invested considerable effort in green, sustainable development—an area that has attracted considerable research attention [3, 4]. In addition, the concept of green development has applied to green financial [5], green supply chain [6], green innovation [7] and so on.

Green development is the way China must choose [8]. Green development is not only key to China’s construction of an ecological civilization, but also a new development model emphasizing development quality and benefits, environmental protection, and efficient resource utilization [9]. Although China’s pursuit of green development has produced significant results [9–34], some problems still need to be considered. As a result of economic, social, natural, and historical differences, green development levels (GDLs) and the paths for achieving green development show obvious regional differences [9]. The Yangtze River Economic Belt (YREB) is an important ecological security barrier and energy base in China that has become part of the national strategy for ecological protection and high-quality development [10, 11]. Research on the GDL of the YREB has mainly focused on evaluating GDL and exploring the driving mechanisms at the provincial, urban, or industrial scale [12]. Few studies, however, have considered the spatiotemporal characteristics of GDL in the YREB. Meanwhile, the effect of a single factor e.g., economic development and technological innovation, on GDL has been widely reported, however the interaction between various factors has been few explored and the path for improving GDL lacks systematic demonstration [13–20]. It is important, therefore, to explore the spatiotemporal characteristics as well as the multiple influencing factors and realization paths of GDL. To address the gap in the literatures, first, we constructed three-level evaluation system for the GDL and evaluated the GDL in the YREB using the entropy weight method (EWM) to determine each indicator’s weight value from 2011 to 2019. Second, we employed a fuzzy-set qualitative comparative analysis (fsQCA) based on complexity and configuration theories to explore the paths of GDL in the YREB. Finally, policy recommendations for the government were proposed to promote the green development of the YREB.

In light of the above, this research aimed to explore three questions: (1) What are the temporal and spatial differences in the GDL of 11 provinces and cities in the YREB? (2) What are the core conditions for each province or city to reach a high GDL in the YREB? (3) How will the configuration paths of various influencing factors improve GDL in the YREB, and what are the specific paths? The purpose of this study is to find the key configuration path of the green development using the EWM and fsQCA methods, and to provide policy suggestions of the green development in the YREB.

This study makes both theoretical and practical contributions. Theoretically, by using fsQCA, we break with traditional research that focuses on the net effect of a single variable and provide a new way to explore coupling effects between multiple variables in terms of configuration, thereby expanding the research on green development paths. This approach enables us to examine the relationships between all possible combinations of influencing factors and outcomes for the YREB. Practically, we reveal the paths for achieving high GDL in terms of economic development level (EDL), scientific technological innovation level (STIL), industrial structure (IS), openness level (OL), environmental regulation (ER), and urbanization scale (US) for each province and city in the YREB. This can provide new ideas for exploring the paths toward green, high-quality development in China.

## 2 Literature review

### 2.1 Evaluation of green development

China, the largest developing country, still faces the outstanding problem of balancing economic growth and environmental protection [11]. By now, China has taken various energy-saving and emission reduction measures to build a resource-saving and environment-friendly society [12]. It also set the goal of carbon peak by 2030 and carbon neutral by 2060, which demonstrates China’s determination to achieve high-quality coordinated development featuring green, low-carbon and circular development [13]. In view of this, scholars have paid extensive attention to green development and conducted a large number of relevant studies [14–35]. Here, we mainly summarize and review green development from the following two aspects.

Green development evaluation is performed to determine the GDL of a region. Green development efficiency (GDE) and comprehensive index evaluation are two main methods for measuring GDL. GDE refers to the ability of unit input cost to obtain desired output under the constraints of resources and environment [12]. The higher the GDE, the higher the GDL. Methods for evaluating GDE have included data envelopment analysis (DEA) [14], stochastic frontier analysis [15], super-efficiency DEA [16, 17], and super-efficiency SBM [18]. For example, Feng et al. estimated the global patterns of green development performance and its influencing factors using a green development performance index based on DEA and the panel data of 165 countries [14]. Meanwhile, Liu et al. measured the industrial green development efficiency of 289 cities in China based on a super-efficiency slacks-based measure (SBM) model [17]. In addition, Wu et al. used the super-efficiency SBM-undesirable model to calculate the green total factor productivity of 30 provinces in China [18]. These existing studies provided some enlightenment for our study, however, they only analyzed the relationships between multiple input and multiple output and didn’t explore specific policy suggestions to region green development.

The comprehensive index evaluation method can complicatedly reflect the GDL and help understand various factors affecting green development. The researchers have constructed various comprehensive evaluation index systems for GDL mainly using economic, resource, environment, social, environmental governance, and so on [19–23]. For instance, Yang et al. constructed a comprehensive GDL evaluation index system from societal, economic, and environmental perspectives [19]. Wang et al. employed multi-level urban green development evaluation index system in five aspects, including living environment, pollutant treatment, ecological efficiency, economic growth and innovative potential [20]. Weng et al. formulated a comprehensive evaluation system of regional green development indicators, including economic advancement, resource utilization, ecological environment, social progress and environmental governance [21]. Furthermore, Hou et al. reconstructed the green productivity evaluation indicator system from environmental quality, ecological resources, economic development, healthy labor and energy utilization [22]. In addition, Sun et al. established the evaluation index system and information entropy model to analyze the evolution of green development in China, and found the differences among the 31 provinces [23]. Clearly, the most-used method is EWM combined with other methods, such as expert scoring method [21], correlation-fuzzy rough set [22], and AHP [19]. EWM is an objective weighting method that can be used for multiple objects and indicators. It can significantly avoid interference from human factors, and its evaluation results are highly accurate and objective, and thus better able to explain results. In addition, EWM extracts information from the evaluation system, which is an orderly process. Based on this, EWM can simultaneously analyze spatial and temporal differences in GDL. Specifically, when the time information of the evaluation system is used before the spatial information, the temporal evolution characteristics of green development are analyzed; otherwise, the spatial differentiation characteristics of green development are analyzed. After fully considering the actual situation and research purpose of the YREB, we selected comprehensive index evaluation methods based on EWM to measure the temporal and spatial differences of green development in the YREB.

### 2.2 Influencing factor of green development

GDL is affected by many factors. Studies have investigated these factors in terms of industrial structure (IS) [24], science and technology innovation [25], environmental regulation (ER) [26], government investment [27], technological progress [24], urbanization [27], economic development level (EDL) [14], energy structure [14], foreign direct investment [24], openness degree [27], consumption level [17], population density and size [17], environmental tax [28], energy conservation [29], emission reduction policy [29], human capital [29], industrial agglomeration [30], and resource endowment [24]. It is generally believed that areas with a high EDL have a high GDL. However, Feng proved there existed a U-shaped between green development performance index and economic development level [14]. Jin et al. pointed out that macroeconomic uncertainty had little impact on the GDL of developed cities and coastal cities, but had an obvious inhibitory effect on the GDL of underdeveloped cities [31]. In recent years, the researches about the ER on green development [32], and the joint impact of ER and scientific and technological innovation on green development [25] have gradually increased. Wang et al. found that formal and informal ER had positive and significant impact on green growth [26]. Huang et al. reported that the impact of ER on the cities’ efficiency of YREB took on a U-shaped trend [32]. Yuan et al. stated that ER promoted the improvements of environmental efficiency in the manufacturing industry [33]. Guo et al. demonstrated that ER showed a significantly negative effect on regional green growth performance and technological innovation had a positive impact on regional green growth performance [25]. Overall, the majority of studies employed regression analysis method, such as spatial autoregressive models [24], panel vector autoregressive models [18], multiscale geographical weighted regression [17], spatial regression [21], spatial Durbin models [34] and random-effect panel Tobit models [35].

Traditional regression analysis is mainly suitable for exploring the “net effect” of a single factor. The methods such as cluster analysis and factor analysis can also test configuration relationships, but cannot effectively identify the interdependence, configuration equivalence, and causal asymmetry between conditions. In contrast, fuzzy-set qualitative comparative analysis (fsQCA) can find the configuration relationship and identify other correlations among multiple factors. FsQCA is suitable for small and medium-sized samples, and focuses on the most typical and minimal antecedent configurations of outcome variables [36]. Therefore, our study used fsQCA to analyze the complex effect mechanisms of different factors’ interdependencies on GDL.

**Fig 1:**
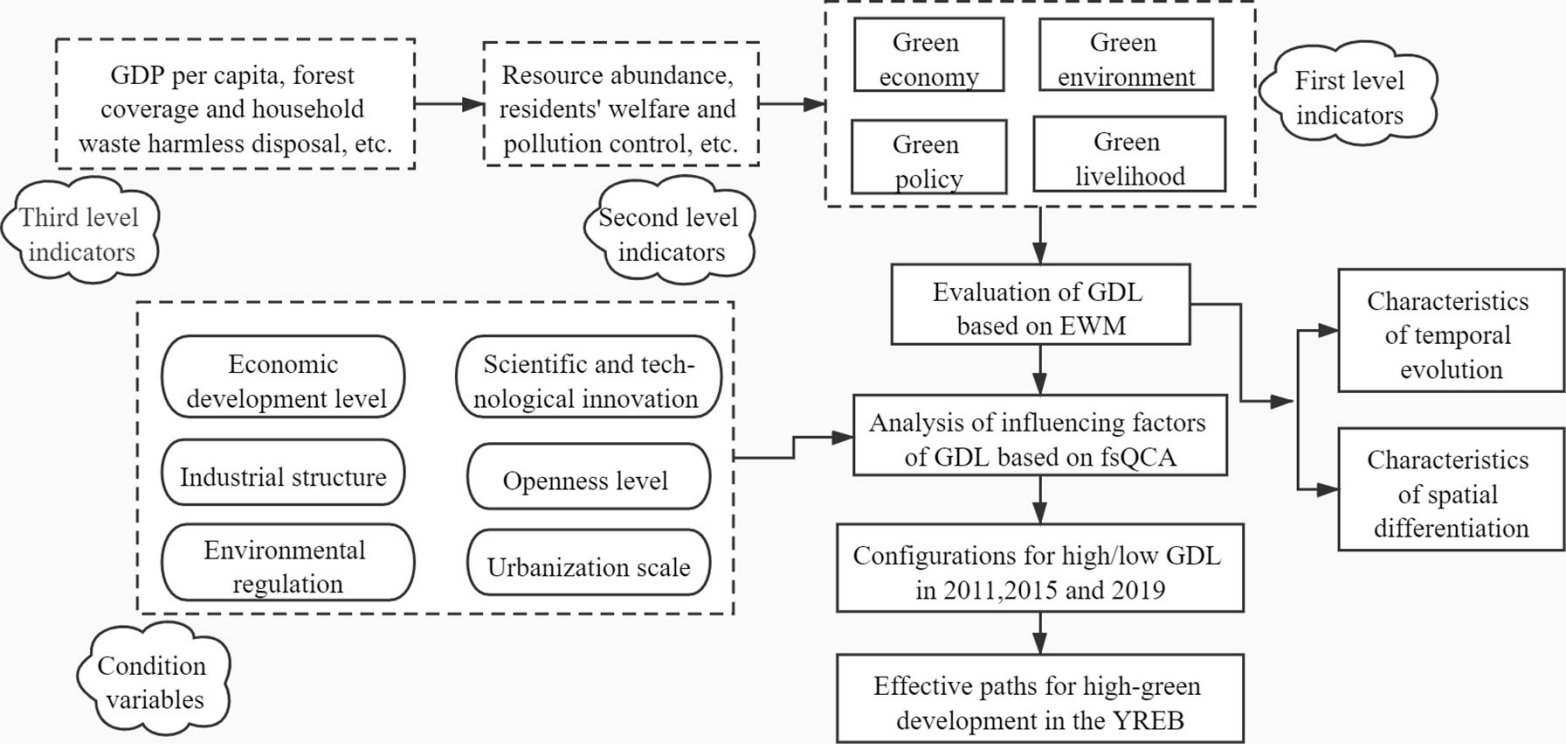
Research framework.

This study constructed an evaluation index system for GDL and analyzed the temporal evolution and spatial differentiation of GDL in the YREB using EWM during 2011–2019. FsQCA was used to study the combined effects of multiple conditional variables on GDL. In this way, the configurations affecting high/low GDL in the YREB in 2011, 2015, and 2019 were obtained. According to the configuration results of influencing factors and the development characteristics of each province, the green development promotion path with regional differentiation is proposed. Fig 1 shows the research framework.

## 3 Materials and methods

### 3.1 Study area

**Fig 2:**
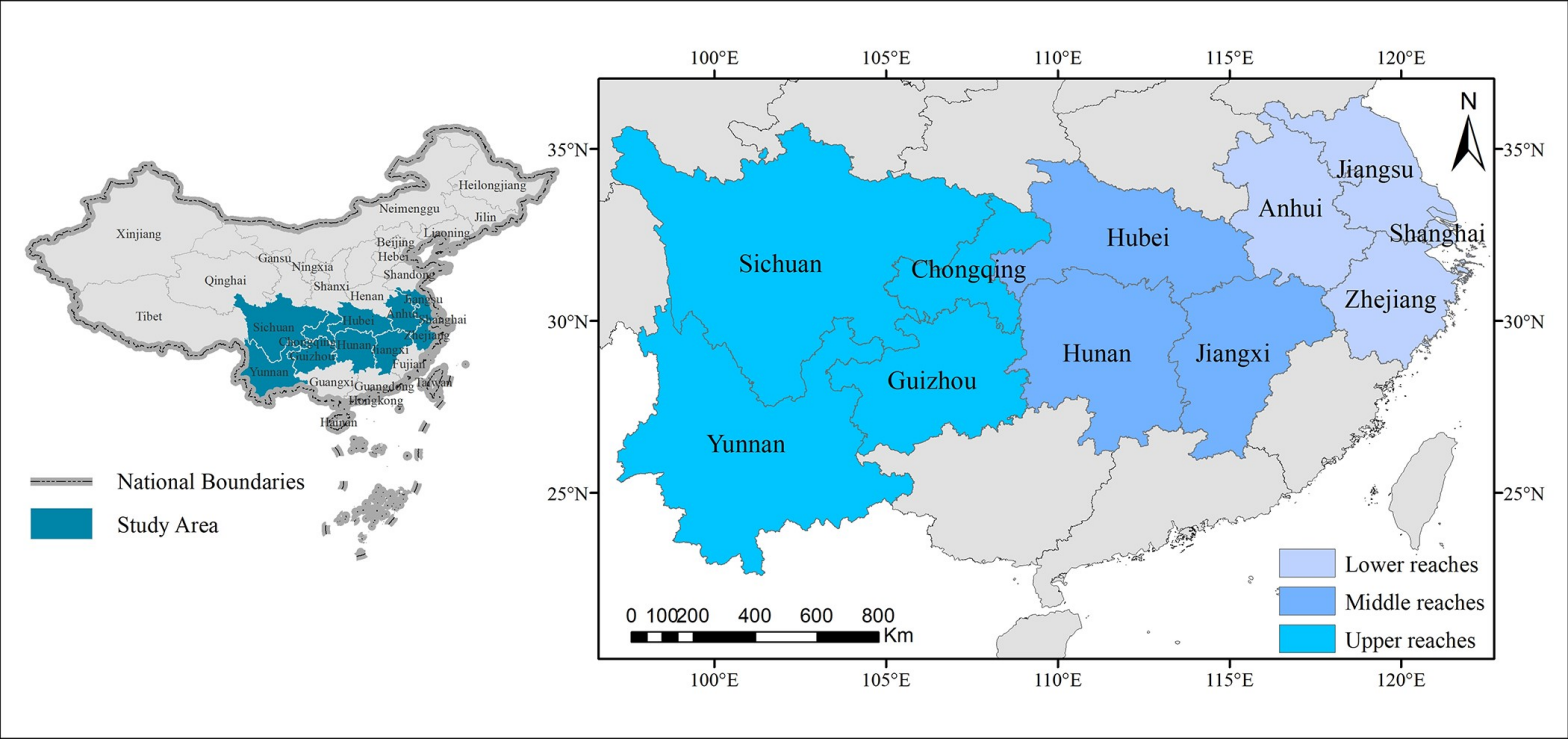
Map of the study area.

The YREB stretches across China’s eastern, central, and western regions. Its total area is about 2.05 million square kilometers, accounting for 20% of China’s total area [37]. It starts from Shanghai in the east and ends in Yunnan in the west, covering nine provinces and two municipalities, and is divided into three regions: the lower reaches (Shanghai, Jiangsu, Zhejiang, and Anhui), middle reaches (Jiangxi, Hubei, and Hunan), and upper reaches (Chongqing, Sichuan, Yunnan, and Guizhou) (Fig 2). Developing the YREB is one of China’s five major national strategies, which explore new ways to coordinate ecological prioritization and green development [38]. In 2019, the YREB had a population of 602 million and a GDP of 45.8 trillion yuan, accounting for nearly half the national totals. In addition, the YREB has a good geographical location that is rich in freshwater and agricultural resources, accounting for one-third of China’s food production and water supply. However, with rapid social and economic development, the contradiction between developing and protecting the YREB has become salient. The YREB faces many problems in need of solutions, including the grim environmental situation, bottlenecks in the Yangtze River waterway, unbalanced regional development, the difficulty of industrial transformation, and poor regional cooperation [7].

### 3.2 Methods

#### 3.2.1 Entropy weight method (EWM)

EWM is a commonly used objective weighting method that calculates the entropy value of indicators using information entropy according to the variation degree of the indicators; then, it corrects indicator weights using the entropy value [39]. The weighting process for EWM is as follows [40]:

Step 1: Data standardization

Positive indicators: $r_{ij}=\frac{x_{ij}-x_{jmin}}{x_{jmax}-x_{jmin}}$; negative indicators: $r_{ij}=\frac{x_{jmax}-x_{ij}}{x_{jmax}-x_{jmin}}$

(among them, *i* = 1,2,…,*m*;*j* = 1,2,…,*n*.).

Step 2: If there are **m** objects to be evaluated and **n** indicators to be evaluated, the dimensionless data matrix R is constructed according to Step 1:

$$R={\left(\begin{array}{cccc} r_{11} & r_{12} & \cdots & r_{1n}\\ r_{21} & r_{22} & \ldots & r_{2n}\\ \vdots & \vdots & \ldots & \vdots \\ r_{m1} & r_{m2} & \ldots & r_{\mathrm{mn}} \end{array}\right)}_{m\times n}$$ (1)

where r_ij_ is the evaluation value of the **i-th** object under the **j-th** indicator.

Step 3: *P*_*ij*_ is used to represent the index value of the **i-th** object under the **j-th** index, and the expression is

Pij=rij∑i=1mrij,(i=1,2,…,m;j=1,2,…,n) (2)

Step 4: Solve the entropy value under the **j-th** index:

$$e_{j}=-\frac{1}{\ln m}\sum_{i=1}^{m}P_{ij}\cdot \ln P_{ij},(i=1,2,\ldots ,m;j=1,2,\ldots ,n)$$ (3)

Step 5: Calculate the weight value of the **j-th** index:

wj=1−ej∑j=1n(1−ej)(j=1,2,…,n) (4)

Step 6: The comprehensive evaluation value of GDL in each province from 2011 to 2019 is

$$W_{i}=\sum_{j=1}^{n}w_{j}\cdot P_{ij}\;(i=1,2,\ldots ,m;j=1,2,\ldots ,n)$$ (5)

#### 3.2.2 Fuzzy-set qualitative comparative analysis (fsQCA)

Qualitative comparative analysis (QCA) is an effective way to explore the adequacy relationships between a set of condition variables and outcome variables. QCA can solve the complex problems of multifactor interactions and explore the causal relationships between a combination of influencing factors and outcome variables [41]. From a configuration perspective, QCA is a process for exploring the joint action of multiple variable combination paths and achieving the same result. According to the set form, QCA can be divided into three main types: clear-set qualitative comparative analysis (csQCA), multivalued qualitative comparative analysis (mvQCA), and fuzzy-set qualitative comparative analysis (fsQCA). Among them, the values of csQCA and mvQCA are discrete variables while fsQCA allows for the continuous assignment of variables, which is applicable to the continuous variable of the original sample data. FsQCA can complete truth-table transformation based on fuzzy-set data [42], offering the advantages of both qualitative and quantitative analysis [43]. By screening the consistency and coverage of the configuration, fsQCA obtains the configuration with theoretical explanatory power. The consistency value represents the degree to which the corresponding configuration sample is similar to the original data; coverage degree is the degree to which the final result of the sample is interpreted by the specific configuration [43]. The formula for consistency and coverage is as follows:

$$c\mathrm{on}\mathrm{sistency}(X\leq Y)=\sum \min(x_{i},y_{i})/\sum x_{i}$$ (6)

$$\mathrm{cov}\mathrm{erage}(X\leq Y)=\sum \min(x_{i},y_{i})/\sum y_{i}$$ (7)

where *X*_*i*_ is the calibrated condition variable, representing the membership degree of sample *i* in combination *X*, and *Y*_*i*_ is the calibrated result, representing the membership degree of sample *i* in result *Y*. Consistency and coverage can be selected from [0,1]. The closer the consistency value is to 1, the greater the correlation between the combination of conditional variables and the sample data. The closer the coverage is to 1, the more easily the conditional variable can explain the result.

FsQCA has been used in many fields, such as national innovation [44], environmental resource management [45], environmental governance [46], and willingness to pay for green energy [47]. The analysis steps of this method mainly include the following [48]: the research object is determined, the condition variables and outcome variables are designed, the initial data of the variables are calibrated, the necessity test of a single variable is conducted, truth tables are constructed, and the configuration is minimized.

### 3.3 Variables

#### 3.3.1 Condition variables

Referring to the literature on analyzing the factors affecting GDL, we selected the following condition variables: economic development level (EDL), scientific and technological innovation level (STIL), industrial structure (IS), openness level (OL), environmental regulation (ER), and urbanization scale (US); the outcome variable was the GDL in the YREB. Table 1 shows the initial information about these variables.

**Table 1:** Initial information about the variables.

Variable Type	Influencing Factor	Abbrevia-tion	Unit	Assignment Instructions
Condition variables	Economic development level	EDL	Yuan/ person	Per capita GDP
	Scientific technological innovation level	STIL	Number	Number of authorized patents
	Industrial structure	IS	%	Proportion of secondary industry
	Openness level	OL	Hundred million dollars	Actual amount of foreign direct investment
	Environment regulation	ER	%	Comprehensive utilization rate of industrial solid waste
	Urbanization scale	US	%	Proportion of permanent urban population in the total population of the region
Outcome variable	Green development level	GDL	——	Based on MATLAB, EWM is used to solve the problem

*(Ⅰ) Economic development level (EDL)*. EDL refers to the scale, speed, and level of a country’s or region’s economic development. Common indicators that reflect EDL include gross national product, per capita income, economic development rate, and economic growth rate. The higher the EDL, the more solid the material foundation for green development [49], thus playing a significant role in promoting green development in the YREB. Per capita GDP can reflect the level of regional economic development to a certain extent; thus, it was chosen to describe the level of economic development [4].

*(Ⅱ) Scientific technological innovation level (STIL)*. STIL is a source of economic growth and an important driving force for economic development [31]. The main indicators for measuring STIL include R&D investment, patent applications, and patent authorizations. Compared with the number of patent applications, the number of authorized patents can more effectively reflect the actual innovation level of a region. Therefore, we took STIL as one factor affecting the GDL of the YREB and denoted it by “the number of patents authorized” [17, 50].

*(Ⅲ) Industrial structure (IS)*. IS is the core driving force of economic development, and it has an important effect on GDL [51]. An increase in the proportion of secondary industry means an increase in pollution. Therefore, IS was included in the factors affecting the GDL of the YREB and was expressed as “the proportion of the secondary industry” [51].

*(Ⅳ) Openness level (OL)*. Foreign direct investment reflects the degree of regional openness and the mobility of production factors. The advanced technologies introduced by foreign investment promote the development of regional innovation. OL has thus become an important factor affecting regional GDL [49]. In view of this, OL was expressed in terms of “the actual amount of direct foreign investment” [29].

*(Ⅴ) Environmental regulation (ER)*. Strict ER is positively related to green production efficiency. Some studies have used the comprehensive utilization rate of industrial solid waste, the standard rate of industrial wastewater discharge, and the removal rate of sulfur dioxide to measure the degree of ER. Considering data availability, the comprehensive utilization rate of industrial solid waste was used in this study to reflect ER [52].

*(Ⅵ) Urbanization scale (US)*. US is an important factor affecting regional economic efficiency, and the interaction between agglomeration and crowding effects will affect regional GDL. US as reflected by the urbanization rate of the resident population (urban resident population/total population) is suited to the reality of urbanization in China [53].

#### 3.3.2 Outcome variable

The measurement of GDL is a complex system involving many factors and indicators [28]. This study determined the GDL of the YREB as the outcome variable. Based on an evaluation system that employed EWM and MATLAB software, we obtained the data for GDL and considered the effective combination path of the six abovementioned condition variables to form the causal relationship.

#### 3.3.3 Evaluation index system

A three-level evaluation system for the GDL was constructed, including 4 first-level indicators, 10 second-level indicators, and 26 third-level indicators (Table 2).

**Table 2:** Evaluation indexes of GDL in the YREB.

First-level index	Second-level index	Third-level index	Unit	Attribute
Green economy (A)	Benefits of economic growth (A_1_)	Per capita GDP (A_11_)	Yuan	+
		Electricity consumption per unit of GDP (A_12_)	Kilowatt hour	-
	Primary industry (A_2_)	Proportion of primary industry in GDP (A_21_)	%	+
		Ratio of effective irrigated area to cultivated area (A_22_)	%	+
	Secondary industry (A_3_)	Proportion of secondary industry in GDP (A_31_)	%	-
	Tertiary industry (A_4_)	Proportion of tertiary industry in GDP (A_41_)	%	+
		Proportion of employed persons in tertiary industry (A_42_)	%	+
Green environment (B)	Abundance of resources (B_1_)	Amount of water per capita (B_11_)	m^3^	+
		Area of cultivated land per capita (B_12_)	ha	+
		Proportion of area covered by nature reserves (B_13_)	%	+
		Forest coverage (B_14_)	%	+
	Environ-mental carrying capacity (B_2_)	Industrial wastewater emissions per capita (B_21_)	t	-
		Per capita emissions of SO_2_ (B_22_)	t	-
		Amount of chemical fertilizer applied per unit cultivated area (B_23_)	Ten t/ha	-
		Nitrogen oxide emissions per capita (B_24_)	t	-
Green livelihood (C)	Welfare of residents (C_1_)	Ratio of disposable income gap between urban and rural residents (C_11_)	Yuan	-
		Number of health facilities per person (C_12_)	-	+
		Proportion of education expenditure in the general budget expenditure of local finance (C_13_)	%	+
	Public service (C_2_)	Public transport vehicles per 10,000 people (C_21_)	-	+
		Green coverage of the built-up area (C_22_)	%	+
		Harmless disposal of domestic garbage (C_23_)	%	+
Green policy (D)	Investment growth (D_1_)	Proportion of expenditure on science and technology in the local general public budget (D_11_)	%	+
		Intensity of R&D expenditure (D_12_)	%	+
		Proportion of the expenditure on environmental protection in budget of local finance (D_13_)	%	+
	Pollution control (D_2_)	Proportion of investment in environmental pollution control in GDP (D_21_)	%	+
		Comprehensive utilization rate of industrial solid waste (D_22_)	%	+

### 3.4 Data

Data collection covered 11 provinces and cities in the YREB from 2011 to 2019, based on the data platform of the Easy Professional Superior (EPS) and the statistical yearbooks [54]. The statistical yearbooks included the *China Urban Statistical Yearbook* [55], the *China Environmental Statistical Yearbook* [56], and the statistical yearbooks for each province and city. Data processing was divided into three steps. First, small amounts of missing data were supplemented in the form of “the current year’s data equals the average value of data in the last five years,” following the principle of proximity. Second, SPSS software was used for the statistical analysis of all data and to calculate the average value of the condition variables during the study period. Based on the constructed GDL evaluation index system, MATLAB was used to compute the value of GDL according to the steps of EWM. Finally, after the calibration of variables and necessity testing for single variables, the data were imported into fsQCA 3.0 to analyze the influence path of GDL in the YREB.

## 4 Results

### 4.1 Comprehensive GDL evaluation results

We examined the temporal evolution and spatial differentiation characteristics of the GDL of the YREB. Temporal information precedes spatial information when discussing the temporal change trend of a research object. The opposite is the case when considering spatial trends. Thus, EWM was used to calculate the GDL of each subsystem, and then the comprehensive GDL was calculated. Moreover, the average value comparison method was used to classify GDL into low-level regions and high-level regions.

#### 4.1.1 Characteristics of time-series evolution

**Fig 3:**
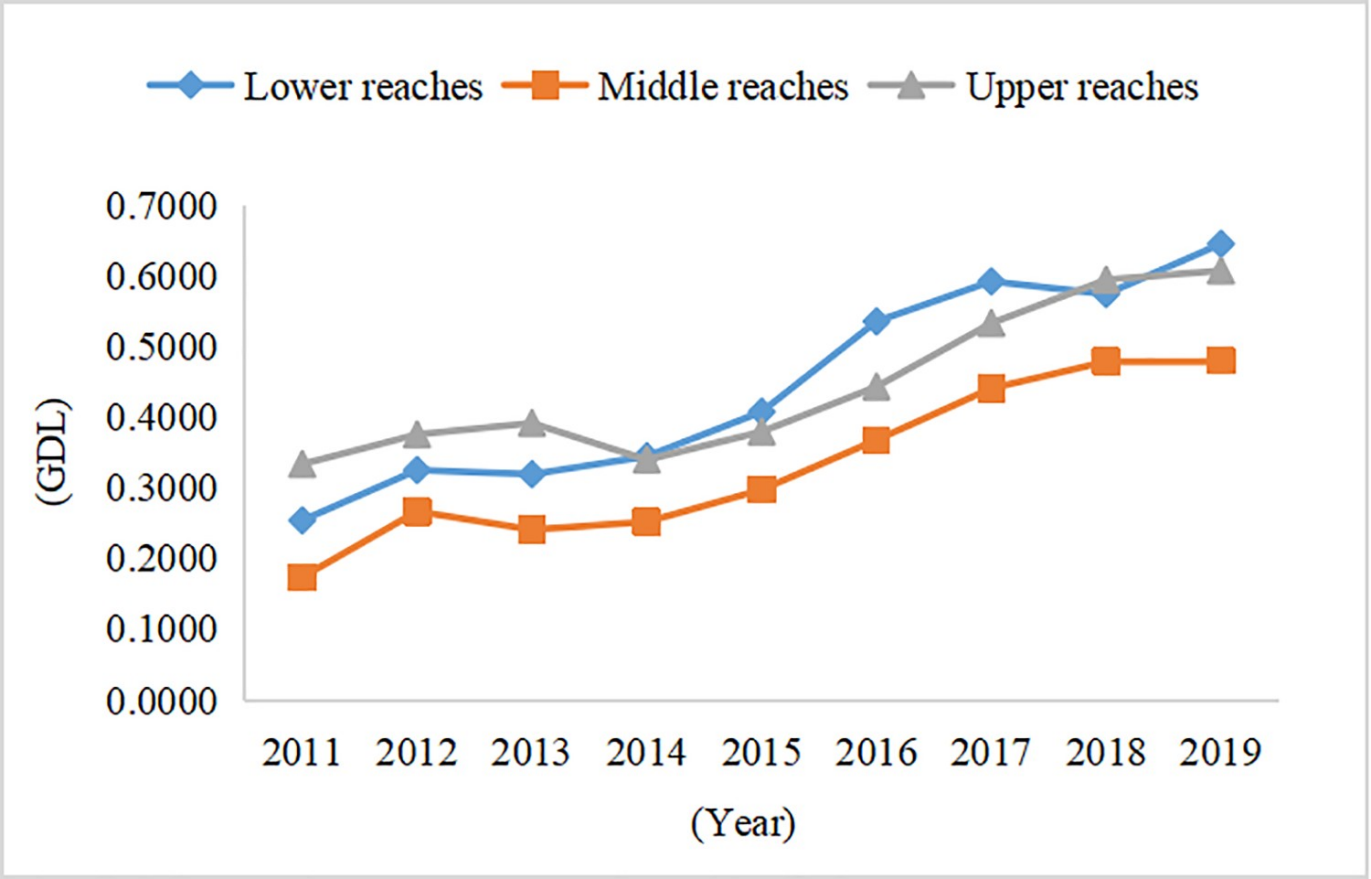
Time series evolution of the GDL in the YREB.

The GDL of most provinces and cities continuously improved during 2011–2019. Anhui, Hunan, Jiangsu, and Zhejiang had the highest average annual growth rates, followed by Hubei, Jiangxi, Shanghai, Guizhou, and Sichuan. Meanwhile, Yunnan and Chongqing had the lowest average annual growth rates. The YREB showed high GDL after 2016, when China proposed its major development strategy for the YREB. The overall GDL of the YREB showed an increasing trend during the study period, with an average annual growth rate of 15.94%. Specifically, the upper reaches increased from 0.3338 in 2011 to 0.6076 in 2019, with an average annual growth rate of 10.25%. The middle reaches increased from 0.1739 in 2011 to 0.4806 in 2019, with an average annual growth rate of 22.05%. In the lower reaches, the growth rate increased from 0.2542 in 2011 to 0.6456 in 2019, with an average annual growth rate of 19.25% (Appendix Table A1 in S1 File). The GDL of the upper reaches was the highest, followed by the lower reaches, and the middle reaches had the lowest GDL before 2014. However, during 2014–2019, the GDL of the lower reaches was the highest, followed by the middle reaches (Fig 3).

**Fig 4:**
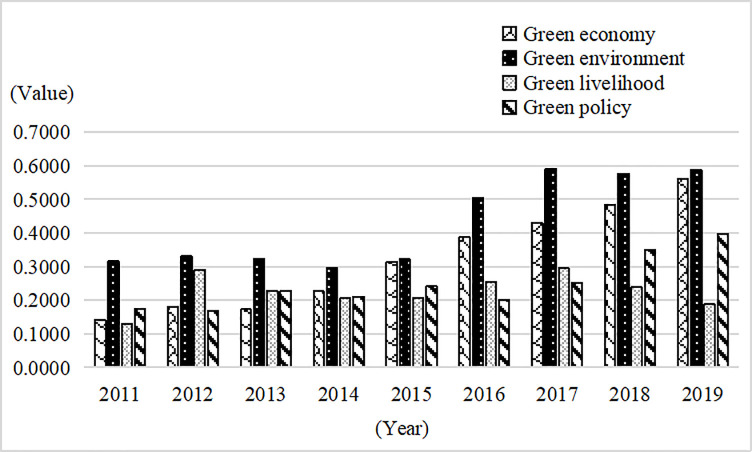
Evaluation values for overall green development in the YREB.

In the composition of GDL in the YREB, the green environment had the highest evaluation value, growing at a rate of 7.13% during 2011–2019, indicating that China actively promoted nature reserves, forest construction, and emission reduction. The green economy value ranked second after 2014, with a growth rate of 16.59%. This indicates that industrial transformation developed in an orderly way, and resource consumption per unit of GDP continued to decline (Appendix Table A2 in S1 File). The gap between green economy and green environment narrowed to the minimum level in 2015. The green environment value was 0.0258 higher than the green economy one in 2019. The value for green policy showed fluctuating growth, with a growth rate of 9.56%, indicating that China continuously increased investment in environmental governance and strengthened pollution control. In Fig 4, in the proportions of the four green indicators of the YREB, green environment is higher than green economy, while green livelihood and green policy are lower than green economy.

#### 4.1.2 Characteristics of spatial differentiation

**Fig 5:**
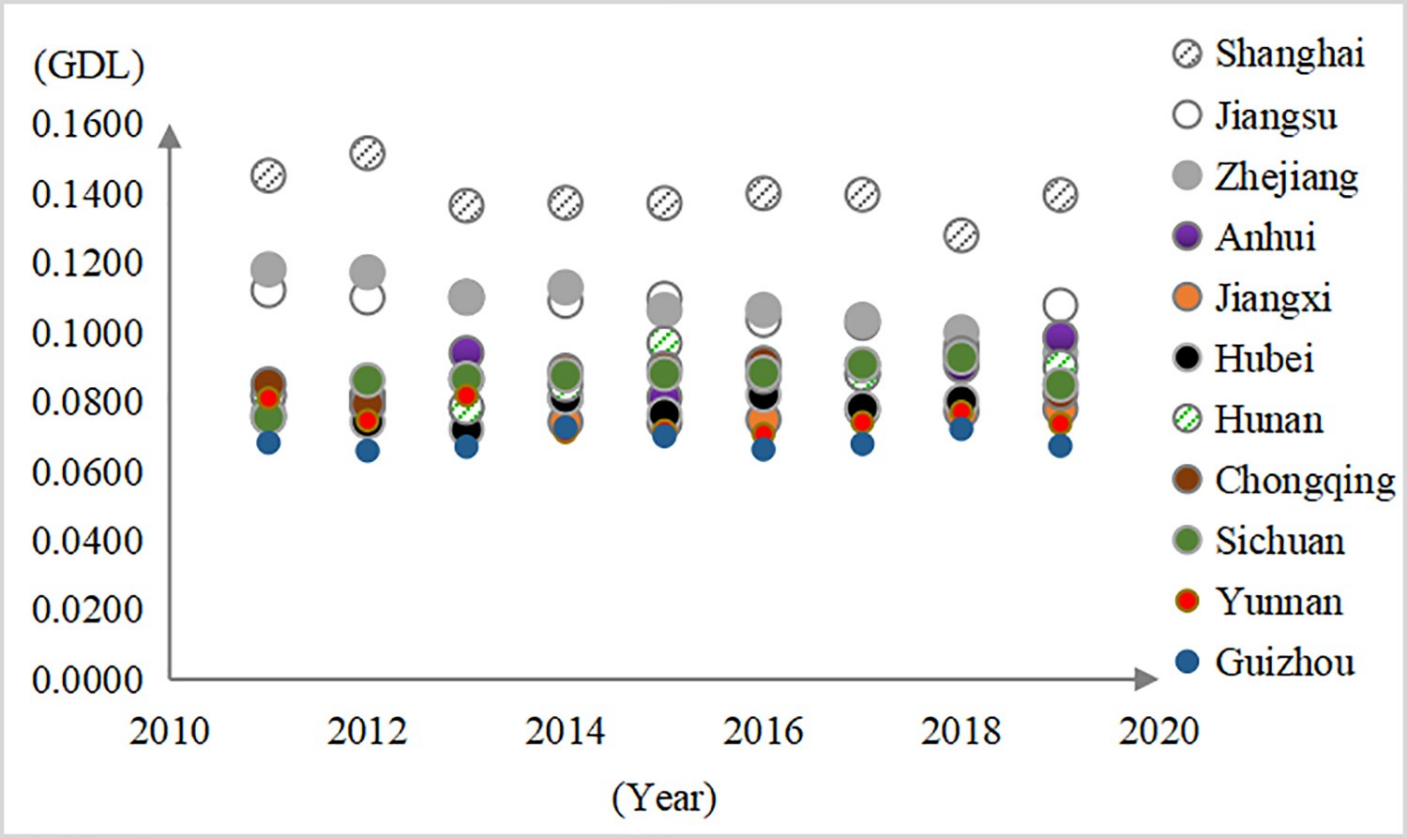
Spatial differences in the GDL in the YREB.

The average GDL of the YREB was 0.0909 during 2011–2019; thus, 0.0909 was taken as the judgment standard. If the GDL of each province and city was greater than or equal to 0.0909, it was considered high GDL; if it was less, it was considered low. The results showed that Shanghai, Jiangsu, and Zhejiang had high GDL (Appendix Table A3 in S1 File), which is consistent with Zeng and Niu [27]. Hunan, Chongqing, and Sichuan in the upper and middle areas showed slightly above average levels in some years (Fig 5). We can see that the overall GDL of the YREB was not high, and the proportion of low GDL was basically stable at over 63%; thus, the regional clustering of low GDL appeared.

In terms of the highest evaluation values in 2019, green economy was the highest in Shanghai, Jiangsu, Zhejiang, Hunan, and Chongqing, followed by green policy in Shanghai, Jiangsu, and Zhejiang and green environment in Hunan and Chongqing. Meanwhile, green environment had the highest value in Yunnan, Hubei, Guizhou, Jiangxi, and Sichuan, followed by green economy and green policy (Table 3); this was because those provinces are rich in ecological resources and have strong ecological carrying capacity. Moreover, Guizhou and Jiangxi have been designated as national ecological civilization pilot zones. The proportion of green policy was the highest in Anhui, mainly because it lacks advantages in economy and natural resources. However, its geographical advantage in the lower reaches of the YREB means it is better supported by the government. In summary, the development modes of the provinces and cities in the YREB are still in the development stage, and the road to improvement will be long and difficult.

**Table 3:** Evaluation values of the GDL of different provinces and cities in 2019.

Province/city	Green economy	Green environment	Green livelihood	Green policy	GDL
Shanghai	0.0732	0.0168	0.0080	0.0413	0.1394
Jiangsu	0.0417	0.0211	0.0213	0.0236	0.1077
Zhejiang	0.0361	0.0156	0.0181	0.0241	0.0939
Anhui	0.0263	0.0207	0.0132	0.0382	0.0983
Jiangxi	0.0156	0.0312	0.0142	0.0168	0.0778
Hubei	0.0232	0.0276	0.0138	0.0202	0.0848
Hunan	0.0286	0.0273	0.0190	0.0151	0.0899
Chongqing	0.0268	0.0234	0.0139	0.0184	0.0825
Sichuan	0.0201	0.0351	0.0182	0.0114	0.0848
Yunnan	0.0193	0.0288	0.0130	0.0125	0.0736
Guizhou	0.0160	0.0234	0.0123	0.0155	0.0672

### 4.2 Path identification based on the coupling effects of combined factors

Based on our assessment of GDL, 2011, 2015, and 2019 were selected as the research period. FsQCA was then used to explore the coupling effects of different combinations of condition variables on GDL. After comparative analysis, an effective path for future green development in the YREB is proposed.

#### 4.2.1 Calibration of variables

Prior to fsQCA, a variable is converted into set membership degree, including the three qualitative anchor points of complete subjection (CSP), intersection point (IP), and complete dissubjection point (CDP), to determine the orientation of the case after assignment [57]. Based on previous research, the upper and lower quartiles were selected as CSP and CDP, respectively, and the average values of the upper and lower quartiles were selected as IP. Table 4 shows the values of the three anchor points determined after the variable transformation. Next, the assignment of the original variable is calibrated and the data are standardized into a value between “0” and “1” to facilitate analysis of the relationship between the configuration of condition variables and the outcome variables.

**Table 4:** Three qualitative anchor points of each variable.

Var. Year		EDL	STIL	IS	OL	ER	US	GDL
2011	CSP	59249	47960	54.3	2019	91.07	61.90	0.1120
	IP	42454	26755	48.4	1174	71.89	51.87	0.0938
	CDP	25659	5550	42.5	329	76.86	41.83	0.0756
2015	CSP	77644	64953	46.0	2918	92.55	65.80	0.1063
	IP	56820	44557	42.9	1720	74.79	56.75	0.0901
	CDP	35997	24161	39.8	521	57.03	47.69	0.0739
2019	CSP	107624	100587	42.6	3388	93.80	70.00	0.0983
	IP	80394	72230	39.4	2080	70.09	61.90	0.0881
	CDP	53164	43872	36.1	772	46.37	53.79	0.0778

#### 4.2.2 Necessity testing for single variables

When the consistency of a condition variable is greater than or equal to 0.9, it is called a necessary condition. Table 5 lists the test results for whether the six condition variables can be judged as necessary conditions for high/low GDL. For example, taking high GDL in 2011, the consistency of EDL, STIL, ER, and US was greater than 0.9, indicating that they were necessary conditions for high GDL that year. In 2015, no variable had a consistency greater than 0.9, meaning there were no necessary conditions for high GDL. In 2019, the consistency of ER was equal to 0.902, indicating that only ER was necessary for high GDL. Based on this, we need to further explore the influence of the combination of condition variables on outcome variables.

**Table 5:** Necessity testing for single condition variables.

Condition variables	Outcome variable (high GDL)			Outcome variable (low GDL)		
	2011	2015	2019	2011	2015	2019
EDL	0.940	0.744	0.681	0.099	0.167	0.212
~EDL	0.219	0.415	0.459	0.980	0.961	0.914
STIL	0.962	0.820	0.837	0.280	0.389	0.325
~STIL	0.211	0.339	0.324	0.805	0.740	0.820
IS	0.633	0.789	0.672	0.646	0.696	0.504
~IS	0.501	0.362	0.459	0.420	0.426	0.613
OL	0.896	0.716	0.630	0.078	0.119	0.104
~OL	0.211	0.413	0.461	0.976	0.984	0.976
ER	0.956	0.789	0.902	0.369	0.327	0.344
~ER	0.225	0.348	0.290	0.721	0.782	0.829
US	0.962	0.779	0.701	0.235	0.259	0.328
~US	0.222	0.366	0.455	0.856	0.858	0.812

Note: The sign “~” in front of a variable indicates that the current variable does not belong to the target set.

#### 4.2.3 Configuration influence path analysis

A consistency threshold value of 0.75 and a case threshold value of 1 were set to conduct configuration minimization calculation in fsQCA3.0 and generate the parsimonious solution, intermediate solution, and complex solution; among these, the former was included in the operation of all logical remainder items (there is no configuration corresponding to the fact case). The intermediate solution is included in the operation of some meaningful logical remainder and is generally considered superior while the latter does not include any operation of logical remainder. Rihoux et al. [57] noted that the first two solutions are mainly observed in QCA analysis cases and stipulated that when an antecedent condition occurs in both the parsimonious and the intermediate solutions, it is called the core condition. If an antecedent condition occurs only in the intermediate solution, it is called an auxiliary condition. Table 6 shows the configuration details of the three research periods. Four configurations leading to high GDL were generated, and their consistencies were 0.991, 0.988, 0.966, and 0.925. Overall consistencies were 0.991, 0.988, and 0.951, indicating that a sufficient condition for the result had been formed. The configuration effect was significant, and the overall coverage rates were 0.868, 0.646, and 0.779, indicating that the obtained configuration could explain more than half of the cases.

**Table 6:** Configurations of high green development levels.

Condition variables	2011	2015	2019	
	CA	CB	CC1	CC2
EDL	•	•	(•)	(⊗)
STIL	(•)	(•)	•	•
IS				(•)
OL	(•)	(•)	(•)	(⊗)
ER	(•)	(•)	•	•
US	(•)	(•)	(•)	(⊗)
Consistency	0.991	0.988	0.966	0.925
Raw coverage	0.868	0.646	0.597	0.261
Unique coverage	0.868	0.646	0.261	0.182
Solution consistency	0.991	0.988	0.951	
Solution coverage	0.868	0.646	0.779	

•/**⊗** represent the existence and nonexistence of core conditions, respectively. (•)/(⊗) represent the existence and nonexistence of auxiliary conditions, respectively.

In 2011 and 2015, CA/CB-US*ER*OL*STIL*EDL was the effective configuration—that is, the core condition was EDL, and the auxiliary conditions were US, ER, OL, and STIL. This means that cases under the CA/CB configuration could achieve high GDL only under the effects of high EDL, US, OL, STIL, and ER. The cases covered by this configuration in 2011 and 2015 were all in Shanghai, Jiangsu, and Zhejiang, and the proportions of sample cases covered were 86.8% and 64.6%, respectively. In 2019, the effective configurations were CC1 and CC2, which shared two core conditions—STIL and ER—and were divided into two configuration paths leading to high GDL as a result of different auxiliary conditions. Among them, the configuration CC1-US*ER*OL*STIL*EDL indicates that STIL, US, OL, EDL, and ER could bring about high GDL. The cases covered by this configuration included Shanghai, Jiangsu, and Zhejiang, with a coverage rate of 59.7%, of which 26.1% could only be explained by this configuration. The combination of condition variables in the CC1 configuration is consistent with that in the CA/CB configuration, but the core conditions are different. CC2-~US*ER*~OL*IS*STIL*~EDL indicates that under the circumstances of no proper US, poor EDL, and insufficient OL, achieving high GDL would require provinces and cities to improve STIL and form effective ER and IS. Only Anhui province was covered by this configuration, and the coverage rate was 26.1%, of which 18.2% could only be explained by this configuration. The main difference between the CC2 configuration and other configurations had to do with different requirements for EDL.

Table 7 shows the configuration paths for low GDL. The consistencies of single configurations were 0.985, 0.990, 1.00, 0.865, 0.920, 0.933, 0.922, 0.838, and 0.956; overall configuration consistencies were 0.985, 0.862, and 0.872. This indicates that sufficient conditions were formed leading to the results, and the configuration effect was significant. Overall coverage rates were 0.800, 0.902, and 0.883, indicating that the obtained configurations could explain more than half of the cases.

**Table 7:** Configurations of low green development levels.

Condition variables	2011			2015			2019		
	CD1	CD2	CD3	CE1	CE2	CE3	CF1	CF2	CF3
EDL	**⊗**	**⊗**	**⊗**	**⊗**	**⊗**	**⊗**	(⊗)	(⊗)	(⊗)
STIL	(⊗)	(•)	(⊗)	(⊗)	(⊗)	(•)		**⊗**	**⊗**
IS		(•)	(•)		(•)	(•)		(⊗)	(•)
OL	(⊗)	(⊗)	(⊗)	(⊗)	(⊗)	(⊗)	(⊗)	(⊗)	(⊗)
ER	(⊗)		(•)	(⊗)			**⊗**		(•)
US	(⊗)	(⊗)	(•)	(⊗)	(•)	(⊗)	(⊗)	(⊗)	(•)
Consistency	0.985	0.990	1.00	0.865	0.920	0.933	0.922	0.838	0.956
Raw coverage	0.631	0.269	0.208	0.617	0.245	0.367	0.755	0.492	0.226
Unique coverage	0.423	0.079	0.090	0.430	0.057	0.218	0.254	0.047	0.081
Solution consistency	0.985			0.862			0.872		
Solution coverage	0.800			0.902			0.883		

•/**⊗** represent the existence and nonexistence of core conditions, respectively. (•)/(⊗) represent the existence and nonexistence of auxiliary conditions, respectively.

There were six configurations leading to low GDL in 2011, 2015, and 2019: the configuration CD1/CE1-~US*~ER*~OL*~STIL*~EDL, covering cases in Yunnan, Guizhou, Jiangxi, and Hunan; CD2/CE3-~US*~OL*IS*STIL*~EDL, covering cases in Anhui and Sichuan; CD3/CF3-US*ER*~OL*IS*~STIL*~EDL, covering Chongqing; CE2-US*~OL*IS*~STIL*~EDL, covering Chongqing and Hubei; CF1-~US*~ER*~OL*~EDL, covering cases in Yunnan, Sichuan, Jiangxi, Guizhou, and Hubei; and CF2-~US*~OL*~IS*~STIL*~EDL, covering cases in Yunnan, Guizhou, and Hunan. Based on the above configurations and coverage cases, we divided the low-GDL provinces and cities into three categories. The first only includes Anhui province, which requires reasonable IS, improved STIL, and effective ER. The second category includes Hunan, Jiangxi, Sichuan, Yunnan, and Guizhou. These have low economic levels but rich natural resources and strong ecological carrying capacity; therefore, an effective path for high GDL should consider EDL or STIL. The third category includes Chongqing and Hubei, whose development advantages and disadvantages have little to do with ER but mainly depend on OL and STIL.

## 5 Discussion

This study used the EWM to analyze the temporal evolution and spatial differentiation of GDL in the YREB during 2011–2019. The results showed that the GDL in the YREB had steadily increased, but the overall level was still not high, with high GDL mainly concentrated in the lower reaches. Many previous studies supported our finding [27, 58–60]. For example, Zeng et al. stated that the GDL of the YREB increased from 2007 to 2016, and Shanghai, Jiangsu and Zhejiang ranked the top three in most years [27]. Chen et al. demonstrated that the eco-efficiency of the YREB had a fluctuating growth trend, and the provinces in the lower reaches was always higher the eco-efficiency than those in the other areas [58]. Wu and Huang stated the level of industrial green development in the YREB had an increasing trend, and that in lower areas was highest, followed by middle area and lowest in upper areas [59]. Hu et al. proved green development of cities in the YREB had gradually improved, and green development in the lower reaches was significantly higher than those in the middle and upper reaches [60]. Collectively, the provinces and cities in the lower reaches have generally high economic level, strong scientific and technological innovation strength and relatively proper industrial system. Meanwhile, more policy support has been given by government, since these provinces and cities were the key development region of the China. With the implementation of strict environmental regulation, high energy utilization efficiency, strong pollution control level, large investment in energy conservation and emission reduction, the green development in the lower reaches has gradually increased in the past decades.

We presented four configurations of path that lead to high GDL. The core conditions of each configuration changed over time. In 2011 and 2015, the configuration CA/CB took EDL as the core condition. The configuration CA/CB offered a better way to green development. In regions with high EDL, effective ER, moderate OL, and high STIL are the basis, supplemented by a reasonable US. This is consistent with previous findings that EDL was the most decisive factor for spatial differences in the YREB [12, 58]. Huang and Wu stated economic development and ER were the main forces to improve the industrial green development efficiency directly based on spatial Dubin model [12]. Chen et al. showed the difference economic development had always been the most decisive factor for the spatial differences in the urban eco-efficiency of the YREB using geographic detectors [58]. In theory, healthy green development means that the proportion of green economic in total green development is the highest, followed by government support [61]. In addition, Mu et al. pointed out that economic foundation was a necessary and insufficient condition for green development, but regions with good economic development did not necessarily have a high GDL [62].

In contrast, our results indicated that the configurations CC1 and CC2 took STIL and ER as the core conditions in 2019. Compared with the other three configurations, the configuration CC2 provided a completely different path to green development. The improvement of GDL was achieved with effective ER, reasonable US and high STIL, when the change of EDL was neglected. Our study proves the Porter hypothesis [63] and is consistent with prior research results [25, 52, 64–66]. For instances, Guo et al. found that technology innovation had positive impact on regional green growth performance, and ER positively affected regional green growth performance through motivating technology innovation [25]. Liu et al. demonstrated that the relationships among ER, green technological innovation, and eco-efficiency had spatial and temporal heterogeneity. Moderate ER was found to assist in reducing the harmful influence of green technological innovation [52]. Cheng et al. proved scientific and technological innovation, urbanization level and IS were the main factors affecting China’s green development [64]. Meanwhile, Chen et al. found that technological innovation and EDL had relatively greater impact than other factors on urban eco-efficiency in the YREB [65]. Teng et al. indicated that the scientific and technological innovation and green development index of the YREB increased simultaneously during the 2006–2016 [66]. Huang et al. showed that the impact of ER on the urban industrial GDE had a gradient decreasing trend in the lower, upper and middle reaches of the YREB [32].

To sum up, the change in the core condition from EDL to STIL and ER indicates that previous models of low labor cost, high energy consumption, and high environmental cost may no longer support economic development. Scientific and technological innovation forms the basis for the new pattern of socioeconomic development in China, and it provides strong support for establishing a resource-saving, environmentally friendly society. There is a two-way causal relationship between ER and GDL that GDL affects the degree of ER in a region. The higher the GDL, the stronger the degree of ER. In our study, GDL in the lower reaches of the YREB also depends on strict ER, high energy efficiency, high levels of pollution control, large investment in energy conservation, and emission reduction. Our findings provide the various solutions that help explain region with different GDL. This confirms that high GDL may be achieved with the absence of factors that were considered as essential in green development (e.g., EDL).

## 6 Conclusion and suggestions

### 6.1 Conclusion

As one of China’s five major national strategy areas, the YREB has unique resource advantages but still faces many challenges in its future development. This study evaluated GDL in 11 provinces and cities of the YREB using EWM during the period 2011–2019. We analyzed the characteristics of time evolution and spatial differentiation, as well as the configuration paths for high/low GDL using fsQCA. The findings are summarized below.

First, overall GDL in the YREB constantly improved over time. Shanghai, Jiangsu, and Zhejiang—all in the lower reaches—had high GDL. The upper and middle reaches all had low GDL. The driving modes of GDL changed from being environmentally driven and government supported in 2011 to being economically and environmentally driven in 2015 and 2019.

Second, there were two effective configurations for high GDL based on fsQCA. The core conditions for high GDL changed from EDL to STIL and ER. In Shanghai, Jiangsu, and Zhejiang in the lower reaches, high EDL, effective ER, moderate OL, and high STIL are the basis for high GDL, supplemented by appropriate US. Meanwhile, the path ~US*ER*~OL*IS*STIL*~EDL is suitable for Anhui. This shows that provinces with poor economic conditions can improve GDL by implementing effective ER and continuously improving STIL without considering the scale of urbanization or the level of openness.

Finally, there were six effective configurations for low GDL based on fsQCA. We summarize two paths for improving GDL. Path 1, ER*OL*IS*STIL*EDL, is suitable for Chongqing and Hubei. Path 2, US*OL*IS*STIL*~EDL, is suitable for Hunan, Jiangxi, Sichuan, Yunnan, and Guizhou. In other words, reasonable OL, IS, and STIL are the basis for improving GDL. If we choose effective ER as the auxiliary condition, the path must consider improved EDL. If appropriate US is selected as the auxiliary condition, whether EDL is improved will not affect this path’s effect on improving GDL.

In summary, we performed configurational analysis and employed fsQCA, deviating from traditional studies in this area, which have focused on variance-based methods. We also identified multiple solutions explaining the same outcome. The different solutions showed that no single factor is sufficient for exploring GDL; rather, a combination of factors is needed.

### 6.2 Suggestions

Based on the analysis of the spatiotemporal evolution and impact mechanism of the GDL in the YREB, combined with the actual situation of the YREB, we propose some suggestions for the YREB that are in line with the characteristics of each province.

1. Our results showed that there was an obvious space heterogeneity in the GDL of the YREB. The government shall take the spatial layout optimization as important contents. We suggest that the central government increases the special financial transfer of ecological compensation in the key ecological functional areas of the YREB, and strengthen the ecological compensation for green ecological industries in the middle and upper reaches. In addition, the horizontal ecological compensation mechanism for the lower reaches to the middle and upper reaches need be established. Thus, the middle and upper reaches can gradually realize the balanced development of ecological and economic contributions, and share the development achievements and benefits. Moreover, the lower reaches should actively strengthen exchanges and cooperation with middle and upper reaches, promote the diffusion and spillover of STIL, and improve the GDL of the YREB as a whole.
2. We found that the provinces in the lower reaches of Shanghai, Jiangsu, and Zhejiang had developed economy, good industrial foundation, strong industrialization level and advancement STIL. They should focus on the effective implementation of ER and the improvement of STIL, along with good EDL, OL, and US as auxiliary conditions in order to maintain high GDL. While the Anhui province had poor economic and ecological levels, low urbanization rate and STIL. The proportion of environmental protection investment in GDP was the highest in Anhui among the provinces in the lower reaches, and its contribution to the promotion of GDL in Anhui Province was limiting, possibly due to the lack of effective management of the use of funds and the low output benefit of investment. We suggest that the government should implement more environmental protection measures and provide policy support to improve STIL and IS, as well as strengthen the supervision of environmental protection funds.
3. Our data indicated that the provinces in the middle and upper reaches of Hunan, Jiangxi, Yunnan, Guizhou, and Sichuan had low EDL, improper IS and weak STIL, but they had good ecological environments and environmental bearing capacities. Due to their special ecological status, these provinces are recommended to adopt the road of green development driven by scientific and technological innovation and to abandon the development mode of resource consumption and environmental destruction in economic development. Meanwhile, the attraction of the central urban area to the rural population within the city and the population outside the city was less in since the low level of urban economic development leads to the limited number of jobs. Furthermore, they had unreasonable product structure, insufficient comprehensive utilization of resources, and low treatment rate of “three wastes”. Therefore, the middle and upper reaches should optimize IS, enhance STIL and OL, and expand US for auxiliary development, building a multifactor combined strategy to improve the GDL.

In short, the YREB faces many challenges in improving its overall GDL. Full play should be given to the demonstration effects of provinces and cities with high GDL to facilitate the catching up of those with low GDL, which help narrow GDL gaps in the YREB and achieve high overall GDL coordination.

This study has some limitations that need be concerned. In terms of the selection of variables, in existing studies, a medium sample of 10~40 cases and 4~6 variables are usually selected. Only 11 provinces and cities in the YREB were considered in this study, and limited condition variables were selected; this could mean some important variables were missed. In addition, the data in this study were panel data, and some data were missing. Although effective methods were used to complete the data, there still may be differences from the actual data.

## Supporting information

S1 File Appendix. (DOCX)

S2 File (DOCX)
