# Supplementary material for: Exploring the green development path of the Yangtze River Economic Belt using the entropy weight method and fuzzy-set qualitative comparative analysis
Source: PLoS One. 2021 Dec 6;16(12):e0260985. doi: 10.1371/journal.pone.0260985 (PMC8648114; doi:10.1371/journal.pone.0260985)
Supplement: S1 File — (DOCX) [file pone.0260985.s001.docx]

**S Appendix**

**Table A1 Various regions and overall GDL in the YREB**

**based on time-series evolution**

|  | Lower reaches | Middle reaches | Upper reaches | Overall |
| --- | --- | --- | --- | --- |
| 2011 | 0.2542 | 0.1739 | 0.3338 | 0.7620 |
| 2012 | 0.3254 | 0.2669 | 0.3758 | 0.9681 |
| 2013 | 0.3194 | 0.2410 | 0.3918 | 0.9523 |
| 2014 | 0.3448 | 0.2521 | 0.3400 | 0.9369 |
| 2015 | 0.4078 | 0.2976 | 0.3796 | 1.0850 |
| 2016 | 0.5358 | 0.3680 | 0.4434 | 1.3472 |
| 2017 | 0.5925 | 0.4410 | 0.5333 | 1.5668 |
| 2018 | 0.5745 | 0.4788 | 0.5946 | 1.6480 |
| 2019 | 0.6456 | 0.4806 | 0.6076 | 1.7338 |

**Table A2 Assessment of the four green development models**

**in the YREB in each year**

|  | Green economy | Green environment | Green livelihood | Green policy |
| --- | --- | --- | --- | --- |
| 2011 | 0.1410 | 0.3159 | 0.1305 | 0.1746 |
| 2012 | 0.1794 | 0.3308 | 0.2899 | 0.1681 |
| 2013 | 0.1727 | 0.3240 | 0.2278 | 0.2277 |
| 2014 | 0.2264 | 0.2950 | 0.2054 | 0.2101 |
| 2015 | 0.3150 | 0.3217 | 0.2069 | 0.2414 |
| 2016 | 0.3873 | 0.5046 | 0.2540 | 0.2013 |
| 2017 | 0.4306 | 0.5902 | 0.2948 | 0.2512 |
| 2018 | 0.4828 | 0.5766 | 0.2383 | 0.3503 |
| 2019 | 0.5612 | 0.5870 | 0.1886 | 0.3970 |

**Table A3 The GDL of provinces and cities in the YREB from 2011 to 2019**

|  | 2011 | 2012 | 2013 | 2014 | 2015 | 2016 | 2017 | 2018 | 2019 |
| --- | --- | --- | --- | --- | --- | --- | --- | --- | --- |
| Shanghai | 0.1450 | 0.1515 | 0.1364 | 0.1372 | 0.1371 | 0.1399 | 0.1395 | 0.1278 | 0.1394 |
| Jiangsu | 0.1120 | 0.1099 | 0.1101 | 0.1089 | 0.1096 | 0.1034 | 0.1026 | 0.0962 | 0.1077 |
| Zhejiang | 0.1180 | 0.1172 | 0.1099 | 0.1129 | 0.1063 | 0.1063 | 0.1035 | 0.0999 | 0.0939 |
| Anhui | 0.0815 | 0.0783 | 0.0939 | 0.0809 | 0.0811 | 0.0904 | 0.0879 | 0.0903 | 0.0983 |
| Jiangxi | 0.0756 | 0.0819 | 0.0781 | 0.0741 | 0.0739 | 0.0748 | 0.0775 | 0.0771 | 0.0778 |
| Hubei | 0.0766 | 0.0741 | 0.0719 | 0.0811 | 0.0762 | 0.0819 | 0.0781 | 0.0801 | 0.0848 |
| Hunan | 0.0820 | 0.0815 | 0.0783 | 0.0849 | 0.0967 | 0.0872 | 0.0878 | 0.0938 | 0.0899 |
| Chongqing | 0.0849 | 0.0793 | 0.0864 | 0.0888 | 0.0895 | 0.0910 | 0.0905 | 0.0931 | 0.0825 |
| Sichuan | 0.0754 | 0.0861 | 0.0865 | 0.0875 | 0.0879 | 0.0882 | 0.0907 | 0.0926 | 0.0848 |
| Yunnan | 0.0809 | 0.0745 | 0.0817 | 0.0711 | 0.0716 | 0.0706 | 0.0740 | 0.0770 | 0.0736 |
| Guizhou | 0.0681 | 0.0658 | 0.0669 | 0.0725 | 0.0700 | 0.0662 | 0.0678 | 0.0721 | 0.0672 |
