# Supplementary material for: Exploring the green development path of the Yangtze River Economic Belt using the entropy weight method and fuzzy-set qualitative comparative analysis
Source: PLoS One. 2021 Dec 6;16(12):e0260985. doi: 10.1371/journal.pone.0260985 (PMC8648114; doi:10.1371/journal.pone.0260985)
Supplement: S2 File — (DOCX) [file pone.0260985.s002.docx]

**The initial value of each index of the evaluation system**

| **Shanghai** | | | | | | | | | |
| --- | --- | --- | --- | --- | --- | --- | --- | --- | --- |
|  | 2011 | 2012 | 2013 | 2014 | 2015 | 2016 | 2017 | 2018 | 2019 |
| A11 | 82560 | 85373 | 90993 | 97370 | 103796 | 116562 | 126634 | 134982 | 157279 |
| A12 | 0.070 | 0.067 | 0.065 | 0.058 | 0.056 | 0.053 | 0.050 | 0.048 | 0.041 |
| A21 | 0.7 | 0.633 | 0.600 | 0.5 | 0.437 | 0.389 | 0.362 | 0.320 | 0.300 |
| A22 | 106.397 | 105.739 | 97.926 | 97.821 | 99.162 | 99.481 | 99.562 | 99.562 | 99.582 |
| A31 | 41.300 | 38.920 | 37.200 | 34.700 | 31.807 | 29.832 | 30.460 | 29.780 | 27.000 |
| A41 | 58.000 | 60.447 | 62.2 | 64.800 | 67.756 | 69.779 | 69.179 | 69.900 | 72.700 |
| A42 | 56.321 | 56.463 | 61.293 | 61.799 | 62.854 | 63.820 | 65.545 | 66.296 | 64.063 |
| B11 | 89.100 | 143.4 | 116.900 | 194.800 | 264.824 | 252.330 | 140.560 | 159.851 | 199.10 |
| B12 | 79.932 | 79.076 | 77.847 | 77.576 | 78.592 | 78.843 | 79.239 | 79.043 | 78.913 |
| B13 | 5.200 | 5.200 | 5.200 | 5.300 | 5.300 | 5.30 | 5.300 | 5.300 | 5.300 |
| B14 | 9.410 | 9.410 | 10.740 | 10.740 | 10.740 | 10.740 | 10.740 | 14.040 | 14.040 |
| B21 | 19.014 | 19.479 | 18.810 | 18.112 | 19.436 | 15.124 | 13.063 | 12.023 | 15.552 |
| B22 | 0.010 | 0.010 | 0.009 | 0.008 | 0.007 | 0.003 | 0.001 | 0.006 | 0.006 |
| B23 | 0.064 | 0.058 | 0.057 | 0.054 | 0.052 | 0.048 | 0.046 | 0.044 | 0.039 |
| B24 | 0.019 | 0.017 | 0.016 | 0.014 | 0.012 | 0.007 | 0.008 | 0.011 | 0.011 |
| C11 | 24478.5 | 26950.8 | 29671.0 | 27649.8 | 29756.7 | 32171.3 | 34770.7 | 37658.9 | 40420.1 |
| C12 | 38.700 | 46.100 | 47.300 | 48.400 | 50.800 | 53.400 | 55.700 | 57.400 | 53.140 |
| C13 | 0.140 | 0.155 | 0.150 | 0.141 | 0.124 | 0.122 | 0.116 | 0.110 | 0.122 |
| C21 | 11.790 | 11.910 | 12.110 | 11.967 | 12.360 | 12.700 | 13.940 | 9.040 | 12.001 |
| C22 | 38.200 | 38.300 | 38.400 | 38.430 | 38.500 | 38.600 | 39.100 | 36.240 | 38.174 |
| C23 | 61.040 | 83.590 | 90.580 | 100.000 | 100.000 | 100.000 | 100.000 | 100.000 | 100.000 |
| D11 | 0.056 | 0.059 | 0.057 | 0.053 | 0.044 | 0.049 | 0.052 | 0.051 | 0.048 |
| D12 | 3.114 | 3.367 | 3.560 | 3.658 | 3.730 | 3.820 | 4.000 | 4.160 | 3.874 |
| D13 | 0.013 | 0.013 | 0.012 | 0.016 | 0.017 | 0.019 | 0.030 | 0.028 | 0.023 |
| D21 | 0.750 | 0.660 | 0.870 | 1.060 | 0.880 | 0.730 | 0.530 | 0.814 | 0.814 |
| D22 | 96.560 | 97.340 | 97.120 | 97.510 | 96.150 | 95.680 | 94.00 | 93.050 | 95.278 |
| **Jiangsu** | | | | | | | | | |
|  | 2011 | 2012 | 2013 | 2014 | 2015 | 2016 | 2017 | 2018 | 2019 |
| A11 | 62290 | 68347 | 75354 | 81874 | 87995 | 96887 | 107150 | 115168.4 | 123607 |
| A12 | 0.087 | 0.085 | 0.082 | 0.077 | 0.073 | 0.071 | 0.068 | 0.066 | 0.063 |
| A21 | 6.20 | 6.323 | 6.20 | 5.60 | 5.685 | 5.269 | 4.711 | 4.47 | 4.30 |
| A22 | 83.219 | 85.713 | 82.62 | 85.053 | 86.395 | 88.689 | 90.348 | 91.396 | 91.955 |
| A31 | 51.30 | 50.172 | 49.20 | 47.40 | 45.702 | 44.735 | 45.016 | 44.50 | 44.40 |
| A41 | 42.40 | 43.505 | 44.70 | 47.00 | 48.613 | 49.997 | 50.274 | 50.98 | 51.30 |
| A42 | 36.10 | 36.50 | 37.00 | 37.70 | 38.60 | 39.30 | 40.30 | 41.10 | 42.10 |
| B11 | 624.60 | 472.00 | 357.60 | 502.30 | 730.531 | 928.58 | 490.27 | 470.638 | 287.50 |
| B12 | 580.808 | 578.876 | 577.10 | 574.648 | 573.583 | 571.459 | 569.598 | 568.041 | 566.704 |
| B13 | 4.10 | 4.10 | 3.90 | 3.80 | 3.80 | 3.80 | 3.80 | 3.80 | 3.80 |
| B14 | 10.48 | 10.48 | 15.80 | 15.80 | 15.80 | 15.80 | 15.80 | 15.20 | 15.20 |
| B21 | 31.181 | 29.81 | 27.782 | 25.74 | 25.881 | 22.428 | 18.919 | 17.836 | 22.161 |
| B22 | 0.013 | 0.013 | 0.012 | 0.011 | 0.010 | 0.007 | 0.005 | 0.009 | 0.009 |
| B23 | 0.073 | 0.072 | 0.071 | 0.071 | 0.070 | 0.068 | 0.066 | 0.064 | 0.063 |
| B24 | 0.019 | 0.019 | 0.017 | 0.015 | 0.013 | 0.012 | 0.011 | 0.014 | 0.014 |
| C11 | 18167.0 | 20317.1 | 21609.7 | 19387.8 | 20916.8 | 22546.0 | 24463.7 | 26354.9 | 28380.7 |
| C12 | 49.02 | 42.10 | 46.40 | 49.30 | 51.90 | 55.40 | 58.40 | 61.10 | 55.22 |
| C13 | 0.176 | 0.192 | 0.184 | 0.178 | 0.180 | 0.185 | 0.186 | 0.176 | 0.176 |
| C21 | 13.21 | 13.36 | 14.15 | 15.083 | 15.806 | 16.57 | 17.42 | 15.63 | 16.102 |
| C22 | 42.10 | 42.20 | 42.40 | 42.61 | 42.83 | 42.94 | 42.97 | 43.13 | 42.896 |
| C23 | 93.77 | 95.93 | 97.36 | 98.10 | 100.00 | 99.93 | 100.00 | 100.00 | 100.00 |
| D11 | 0.034 | 0.037 | 0.039 | 0.039 | 0.038 | 0.038 | 0.040 | 0.044 | 0.045 |
| D12 | 2.17 | 2.382 | 2.489 | 2.539 | 2.57 | 2.66 | 2.63 | 2.70 | 2.62 |
| D13 | 0.027 | 0.028 | 0.029 | 0.028 | 0.032 | 0.029 | 0.028 | 0.027 | 0.030 |
| D21 | 1.17 | 1.22 | 1.49 | 1.35 | 1.36 | 0.99 | 0.83 | 1.204 | 1.204 |
| D22 | 94.89 | 91.37 | 96.74 | 96.82 | 95.28 | 91.23 | 93.83 | 93.63 | 94.158 |
| **Zhejiang** | | | | | | | | | |
|  | 2011 | 2012 | 2013 | 2014 | 2015 | 2016 | 2017 | 2018 | 2019 |
| A11 | 59249 | 63374 | 68805 | 73002 | 77644 | 84916 | 92057 | 98643 | 107624 |
| A12 | 0.096 | 0.093 | 0.091 | 0.087 | 0.083 | 0.082 | 0.081 | 0.081 | 0.075 |
| A21 | 4.90 | 4.811 | 4.80 | 4.40 | 4.274 | 4.159 | 3.736 | 3.50 | 3.40 |
| A22 | 73.516 | 74.315 | 71.236 | 72.114 | 72.382 | 73.246 | 73.075 | 72.878 | 71.088 |
| A31 | 51.20 | 49.953 | 49.10 | 47.70 | 45.962 | 44.855 | 42.945 | 41.83 | 42.60 |
| A41 | 43.90 | 45.236 | 46.10 | 47.80 | 49.764 | 50.986 | 53.319 | 54.67 | 54.00 |
| A42 | 34.57 | 34.90 | 36.36 | 36.78 | 38.48 | 40.20 | 41.98 | 43.47 | 43.97 |
| B11 | 1365.70 | 2641.30 | 1697.20 | 2057.30 | 2547.48 | 2378.11 | 1592.07 | 1520.45 | 2281.00 |
| B12 | 362.731 | 361.402 | 359.858 | 358.860 | 357.212 | 353.238 | 349.479 | 344.605 | 337.949 |
| B13 | 1.50 | 1.50 | 1.60 | 1.60 | 1.60 | 1.68 | 1.68 | 1.68 | 1.68 |
| B14 | 57.41 | 57.41 | 59.07 | 59.07 | 59.07 | 59.07 | 59.07 | 59.43 | 59.43 |
| B21 | 33.359 | 32.028 | 29.770 | 27.121 | 26.603 | 23.240 | 21.728 | 20.906 | 23.920 |
| B22 | 0.012 | 0.011 | 0.011 | 0.010 | 0.010 | 0.005 | 0.003 | 0.008 | 0.008 |
| B23 | 0.046 | 0.047 | 0.047 | 0.045 | 0.044 | 0.043 | 0.042 | 0.039 | 0.037 |
| B24 | 0.016 | 0.015 | 0.014 | 0.012 | 0.011 | 0.007 | 0.008 | 0.010 | 0.010 |
| C11 | 21193.7 | 23442.9 | 23747.1 | 21019.4 | 22589.5 | 24371.1 | 26305.0 | 28271.9 | 30306.5 |
| C12 | 61.70 | 38.90 | 41.80 | 44.60 | 49.20 | 51.90 | 55.40 | 57.90 | 51.80 |
| C13 | 0.196 | 0.211 | 0.201 | 0.200 | 0.190 | 0.186 | 0.190 | 0.182 | 0.176 |
| C21 | 13.550 | 13.960 | 14.640 | 15.456 | 15.986 | 16.270 | 16.930 | 16.100 | 16.148 |
| C22 | 38.40 | 39.90 | 40.30 | 40.75 | 40.58 | 41.02 | 40.36 | 41.19 | 40.78 |
| C23 | 96.43 | 98.97 | 99.44 | 100.00 | 99.22 | 99.98 | 100.00 | 100.00 | 100.00 |
| D11 | 0.037 | 0.040 | 0.041 | 0.040 | 0.038 | 0.039 | 0.040 | 0.044 | 0.051 |
| D12 | 1.851 | 2.085 | 2.165 | 2.261 | 2.360 | 2.430 | 2.450 | 2.570 | 2.414 |
| D13 | 0.020 | 0.019 | 0.021 | 0.023 | 0.025 | 0.023 | 0.025 | 0.023 | 0.027 |
| D21 | 0.740 | 1.080 | 1.040 | 1.180 | 1.030 | 1.380 | 0.870 | 1.100 | 1.100 |
| D22 | 91.070 | 90.450 | 93.240 | 92.750 | 92.550 | 89.340 | 89.850 | 92.110 | 93.800 |
| **Anhui** | | | | | | | | | |
|  | 2011 | 2012 | 2013 | 2014 | 2015 | 2016 | 2017 | 2018 | 2019 |
| A11 | 25659 | 28792 | 32001 | 34425 | 35997 | 39561 | 43401 | 47711 | 58496 |
| A12 | 0.080 | 0.079 | 0.079 | 0.076 | 0.075 | 0.074 | 0.071 | 0.071 | 0.062 |
| A21 | 13.200 | 12.658 | 12.300 | 11.500 | 11.164 | 10.520 | 9.558 | 8.790 | 7.900 |
| A22 | 60.268 | 60.958 | 73.184 | 73.767 | 74.926 | 75.628 | 76.773 | 77.355 | 78.080 |
| A31 | 54.300 | 54.641 | 54.600 | 53.100 | 49.746 | 48.434 | 47.518 | 46.130 | 41.300 |
| A41 | 32.500 | 32.701 | 33.000 | 35.400 | 39.091 | 41.046 | 42.925 | 45.080 | 50.800 |
| A42 | 36.800 | 36.800 | 36.800 | 36.800 | 30.000 | 36.800 | 40.100 | 40.300 | 36.800 |
| B11 | 1010.1 | 1172.6 | 974.5 | 1285.4 | 1495.3 | 2018.2 | 1260.8 | 1328.9 | 850.9 |
| B12 | 986.344 | 982.181 | 975.638 | 965.330 | 955.876 | 951.127 | 937.938 | 927.704 | 921.583 |
| B13 | 3.80 | 3.80 | 3.80 | 3.20 | 3.30 | 3.68 | 3.63 | 3.63 | 3.63 |
| B14 | 26.06 | 26.06 | 27.53 | 27.53 | 27.53 | 27.53 | 27.53 | 28.65 | 28.65 |
| B21 | 11.85 | 11.218 | 11.77 | 11.438 | 11.627 | 8.044 | 6.876 | 9.951 | 9.951 |
| B22 | 0.009 | 0.009 | 0.008 | 0.008 | 0.008 | 0.005 | 0.004 | 0.007 | 0.007 |
| B23 | 0.056 | 0.057 | 0.058 | 0.058 | 0.058 | 0.056 | 0.054 | 0.053 | 0.051 |
| B24 | 0.016 | 0.015 | 0.014 | 0.013 | 0.012 | 0.008 | 0.008 | 0.011 | 0.011 |
| C11 | 14518.9 | 16364.1 | 16156.2 | 14922.1 | 16115.0 | 17435.5 | 18882.1 | 20397.1 | 22124.1 |
| C12 | 36.30 | 37.10 | 39.10 | 41.60 | 43.50 | 45.50 | 48.90 | 51.90 | 46.28 |
| C13 | 0.171 | 0.181 | 0.169 | 0.159 | 0.164 | 0.165 | 0.164 | 0.169 | 0.165 |
| C21 | 9.74 | 10.14 | 10.99 | 11.60 | 11.393 | 11.95 | 13.61 | 12.80 | 12.271 |
| C22 | 39.50 | 38.80 | 39.90 | 41.18 | 41.16 | 41.71 | 42.15 | 42.50 | 41.74 |
| C23 | 86.99 | 91.14 | 98.82 | 99.50 | 99.55 | 99.94 | 99.94 | 100 | 100 |
| D11 | 0.023 | 0.024 | 0.025 | 0.028 | 0.028 | 0.047 | 0.042 | 0.045 | 0.051 |
| D12 | 1.403 | 1.637 | 1.831 | 1.888 | 1.960 | 1.970 | 2.050 | 2.160 | 2.006 |
| D13 | 0.025 | 0.024 | 0.025 | 0.022 | 0.024 | 0.024 | 0.032 | 0.032 | 0.042 |
| D21 | 1.75 | 1.92 | 2.66 | 2.06 | 2.00 | 2.04 | 1.84 | 2.12 | 2.12 |
| D22 | 78.70 | 85.39 | 87.64 | 87.22 | 88.48 | 82.62 | 90.90 | 87.372 | 87.372 |
| **Jiangxi** | | | | | | | | | |
|  | 2011 | 2012 | 2013 | 2014 | 2015 | 2016 | 2017 | 2018 | 2019 |
| A11 | 26150 | 28800 | 31930 | 34674 | 36724 | 40400 | 43424 | 47434 | 53164 |
| A12 | 0.071 | 0.067 | 0.066 | 0.065 | 0.065 | 0.064 | 0.065 | 0.065 | 0.062 |
| A21 | 11.90 | 11.74 | 11.40 | 10.70 | 10.602 | 10.295 | 9.173 | 8.60 | 8.30 |
| A22 | 60.535 | 61.849 | 64.639 | 64.873 | 65.776 | 66.084 | 66.086 | 65.846 | 65.979 |
| A31 | 54.60 | 53.615 | 53.50 | 52.50 | 50.297 | 47.73 | 48.125 | 46.62 | 44.20 |
| A41 | 33.50 | 34.644 | 35.10 | 36.80 | 39.101 | 41.975 | 42.702 | 44.84 | 47.50 |
| A42 | 35.50 | 36.10 | 36.50 | 37.00 | 37.50 | 38.30 | 38.90 | 39.60 | 38.26 |
| B11 | 2319.10 | 4836.00 | 3155.30 | 3600.60 | 4394.48 | 4850.62 | 3592.47 | 2479.18 | 4405.40 |
| B12 | 687.455 | 684.614 | 682.729 | 679.304 | 675.142 | 671.211 | 667.676 | 663.941 | 661.380 |
| B13 | 7.10 | 7.60 | 7.50 | 7.70 | 7.30 | 7.34 | 7.33 | 7.33 | 7.33 |
| B14 | 58.32 | 58.32 | 60.01 | 60.01 | 60.01 | 60.01 | 60.01 | 61.16 | 61.16 |
| B21 | 15.864 | 15.069 | 15.088 | 14.279 | 16.735 | 18.625 | 8.915 | 8.511 | 13.413 |
| B22 | 0.013 | 0.013 | 0.012 | 0.012 | 0.012 | 0.006 | 0.005 | 0.009 | 0.009 |
| B23 | 0.046 | 0.046 | 0.046 | 0.046 | 0.047 | 0.046 | 0.044 | 0.040 | 0.037 |
| B24 | 0.014 | 0.013 | 0.013 | 0.012 | 0.011 | 0.009 | 0.008 | 0.010 | 0.010 |
| C11 | 11764.9 | 13320.8 | 13860.6 | 14192.6 | 15361.0 | 16535.6 | 17956.2 | 19359.5 | 20749.6 |
| C12 | 27.30 | 36.40 | 38.50 | 41.10 | 43.30 | 45.50 | 50.60 | 53.70 | 46.84 |
| C13 | 0.187 | 0.206 | 0.191 | 0.183 | 0.180 | 0.184 | 0.184 | 0.186 | 0.180 |
| C21 | 9.78 | 10.01 | 9.15 | 8.564 | 8.687 | 8.86 | 12.55 | 9.72 | 9.676 |
| C22 | 46.80 | 46.00 | 45.10 | 44.61 | 44.09 | 43.63 | 45.22 | 45.92 | 44.694 |
| C23 | 88.27 | 89.05 | 93.28 | 93.10 | 94.46 | 94.97 | 97.56 | 100 | 100 |
| D11 | 0.008 | 0.009 | 0.013 | 0.015 | 0.017 | 0.018 | 0.023 | 0.026 | 0.029 |
| D12 | 0.827 | 0.878 | 0.94 | 0.975 | 1.04 | 1.13 | 1.23 | 1.41 | 1.157 |
| D13 | 0.017 | 0.022 | 0.021 | 0.018 | 0.02 | 0.026 | 0.028 | 0.029 | 0.03 |
| D21 | 2.06 | 2.44 | 1.67 | 1.47 | 1.41 | 1.69 | 1.52 | 1.552 | 1.552 |
| D22 | 55.27 | 54.53 | 55.83 | 56.56 | 57.03 | 38.69 | 37.15 | 42.41 | 46.368 |
| **Hubei** | | | | | | | | | |
|  | 2011 | 2012 | 2013 | 2014 | 2015 | 2016 | 2017 | 2018 | 2019 |
| A11 | 34197 | 38572 | 42826 | 47145 | 50654 | 55665 | 60199 | 66616 | 77387 |
| A12 | 0.080 | 0.074 | 0.073 | 0.068 | 0.063 | 0.060 | 0.058 | 0.053 | 0.048 |
| A21 | 13.10 | 12.803 | 12.60 | 11.60 | 11.201 | 11.203 | 9.947 | 9.01 | 8.30 |
| A22 | 46.321 | 48.183 | 52.849 | 54.266 | 55.169 | 55.394 | 55.753 | 55.996 | 56.705 |
| A31 | 50.00 | 50.305 | 49.30 | 46.90 | 45.697 | 44.862 | 43.525 | 43.41 | 41.70 |
| A41 | 36.90 | 36.892 | 38.10 | 41.50 | 43.102 | 43.935 | 46.528 | 47.58 | 50.00 |
| A42 | 21.00 | 21.199 | 21.501 | 22.628 | 22.799 | 23.039 | 23.241 | 23.464 | 23.034 |
| B11 | 1319.1 | 1411.0 | 1364.9 | 1574.3 | 1740.9 | 2552.61 | 2118.94 | 1450.21 | 1036.30 |
| B12 | 920.719 | 915.383 | 910.812 | 904.694 | 897.984 | 891.30 | 887.14 | 884.891 | 883.398 |
| B13 | 5.20 | 5.10 | 5.50 | 5.50 | 5.60 | 5.70 | 5.72 | 5.72 | 5.72 |
| B14 | 31.14 | 31.14 | 38.40 | 38.40 | 38.40 | 38.40 | 38.40 | 39.61 | 39.61 |
| B21 | 18.137 | 15.852 | 14.656 | 14.040 | 13.810 | 8.342 | 7.482 | 7.749 | 10.284 |
| B22 | 0.012 | 0.011 | 0.010 | 0.010 | 0.009 | 0.005 | 0.004 | 0.003 | 0.006 |
| B23 | 0.067 | 0.067 | 0.067 | 0.066 | 0.064 | 0.063 | 0.061 | 0.056 | 0.052 |
| B24 | 0.012 | 0.011 | 0.011 | 0.010 | 0.009 | 0.007 | 0.006 | 0.008 | 0.008 |
| C11 | 13295.4 | 15052.1 | 15488.7 | 14003.2 | 15207.6 | 16660.8 | 18077.3 | 19476.8 | 21210.5 |
| C12 | 39.40 | 43.80 | 49.70 | 54.60 | 58.60 | 61.30 | 63.70 | 66.50 | 60.94 |
| C13 | 0.152 | 0.195 | 0.158 | 0.157 | 0.149 | 0.163 | 0.162 | 0.147 | 0.144 |
| C21 | 11.17 | 11.25 | 11.56 | 11.909 | 11.838 | 12.76 | 12.38 | 10.98 | 11.973 |
| C22 | 38.40 | 38.90 | 38.10 | 37.87 | 37.47 | 37.60 | 38.43 | 38.37 | 37.948 |
| C23 | 61.02 | 71.51 | 85.40 | 90.20 | 91.51 | 95.80 | 99.89 | 99.978 | 99.984 |
| D11 | 0.014 | 0.014 | 0.018 | 0.027 | 0.026 | 0.030 | 0.034 | 0.037 | 0.040 |
| D12 | 1.645 | 1.728 | 1.800 | 1.867 | 1.900 | 1.860 | 1.920 | 2.090 | 1.927 |
| D13 | 0.031 | 0.025 | 0.025 | 0.021 | 0.024 | 0.023 | 0.021 | 0.029 | 0.035 |
| D21 | 1.320 | 1.280 | 1.020 | 1.160 | 0.840 | 1.420 | 1.190 | 1.126 | 1.126 |
| D22 | 79.08 | 75.38 | 75.74 | 76.68 | 67.78 | 60.94 | 59.32 | 66.09 | 66.162 |
| **Hunan** | | | | | | | | | |
|  | 2011 | 2012 | 2013 | 2014 | 2015 | 2016 | 2017 | 2018 | 2019 |
| A11 | 29880 | 33480 | 36943 | 40271 | 42754 | 46382 | 49558 | 52949 | 57540 |
| A12 | 0.079 | 0.071 | 0.061 | 0.056 | 0.053 | 0.050 | 0.047 | 0.048 | 0.047 |
| A21 | 14.10 | 13.56 | 12.60 | 11.60 | 11.527 | 11.341 | 8.844 | 8.470 | 9.200 |
| A22 | 66.757 | 65.501 | 74.329 | 74.758 | 75.016 | 75.501 | 75.786 | 76.223 | 76.514 |
| A31 | 47.60 | 47.424 | 47.00 | 46.20 | 44.325 | 42.284 | 41.724 | 39.68 | 37.60 |
| A41 | 38.30 | 39.016 | 40.30 | 42.20 | 44.148 | 46.375 | 49.433 | 51.80 | 53.20 |
| A42 | 34.768 | 34.87 | 35.078 | 35.483 | 35.82 | 36.244 | 37.485 | 38.511 | 36.032 |
| B11 | 1711.9 | 3005.7 | 2373.6 | 2680.1 | 2839.15 | 3229.11 | 2795.46 | 1952.01 | 3037.30 |
| B12 | 627.35 | 624.522 | 620.161 | 615.853 | 611.853 | 608.150 | 605.102 | 601.681 | 600.029 |
| B13 | 5.90 | 6.10 | 6.10 | 6.20 | 6.20 | 6.21 | 5.78 | 5.78 | 5.78 |
| B14 | 44.76 | 44.76 | 47.77 | 47.77 | 47.77 | 47.77 | 47.77 | 49.69 | 49.69 |
| B21 | 14.736 | 14.631 | 13.796 | 12.212 | 11.335 | 7.138 | 5.069 | 4.741 | 10.457 |
| B22 | 0.010 | 0.010 | 0.010 | 0.009 | 0.009 | 0.005 | 0.003 | 0.008 | 0.008 |
| B23 | 0.059 | 0.060 | 0.060 | 0.060 | 0.059 | 0.059 | 0.059 | 0.058 | 0.055 |
| B24 | 0.010 | 0.009 | 0.009 | 0.008 | 0.007 | 0.006 | 0.005 | 0.007 | 0.007 |
| C11 | 13516.8 | 15364.4 | 15614.4 | 16510.0 | 17845.5 | 19353.5 | 21012.2 | 22605.7 | 24447.1 |
| C12 | 52.76 | 43.20 | 46.90 | 52.80 | 58.50 | 62.40 | 65.90 | 69.90 | 61.90 |
| C13 | 0.154 | 0.196 | 0.173 | 0.166 | 0.162 | 0.163 | 0.162 | 0.159 | 0.158 |
| C21 | 10.36 | 10.38 | 10.80 | 12.461 | 13.642 | 15.13 | 14.43 | 15.49 | 14.231 |
| C22 | 36.80 | 37.00 | 37.60 | 38.64 | 39.69 | 40.60 | 41.21 | 41.16 | 40.26 |
| C23 | 86.35 | 95.01 | 96.03 | 99.70 | 99.80 | 99.89 | 99.75 | 99.956 | 99.982 |
| D11 | 0.012 | 0.012 | 0.012 | 0.012 | 0.012 | 0.011 | 0.013 | 0.017 | 0.021 |
| D12 | 1.186 | 1.299 | 1.328 | 1.360 | 1.430 | 1.500 | 1.640 | 1.810 | 1.548 |
| D13 | 0.024 | 0.027 | 0.027 | 0.027 | 0.026 | 0.027 | 0.025 | 0.026 | 0.030 |
| D21 | 0.650 | 0.860 | 0.950 | 0.790 | 1.860 | 0.640 | 0.630 | 0.974 | 0.974 |
| D22 | 66.20 | 63.93 | 64.19 | 63.60 | 65.40 | 73.53 | 81.96 | 83.60 | 70.301 |
| **Chongqing** | | | | | | | | | |
|  | 2011 | 2012 | 2013 | 2014 | 2015 | 2016 | 2017 | 2018 | 2019 |
| A11 | 34500 | 38914 | 43223 | 47850 | 52321 | 58502 | 63442 | 65933 | 75828 |
| A12 | 0.072 | 0.063 | 0.064 | 0.061 | 0.056 | 0.052 | 0.051 | 0.055 | 0.049 |
| A21 | 8.40 | 8.239 | 8.00 | 7.40 | 7.318 | 7.346 | 6.569 | 6.77 | 6.60 |
| A22 | 28.285 | 28.679 | 27.494 | 27.593 | 28.274 | 28.986 | 29.296 | 29.409 | 29.441 |
| A31 | 55.40 | 52.37 | 50.50 | 45.80 | 44.978 | 44.525 | 44.194 | 40.90 | 40.20 |
| A41 | 36.20 | 39.392 | 41.40 | 46.80 | 47.704 | 48.129 | 49.236 | 52.33 | 53.20 |
| A42 | 37.20 | 37.80 | 38.60 | 39.90 | 41.40 | 43.40 | 45.40 | 46.90 | 48.00 |
| B11 | 1773.30 | 1626.50 | 1603.90 | 2155.90 | 1518.65 | 1994.72 | 2142.92 | 1697.22 | 1600.10 |
| B12 | 839.226 | 832.36 | 826.869 | 820.662 | 805.602 | 781.660 | 770.667 | 763.959 | 758.579 |
| B13 | 10.30 | 10.30 | 10.30 | 10.03 | 9.90 | 10.04 | 9.63 | 9.63 | 9.63 |
| B14 | 34.85 | 34.85 | 38.43 | 38.43 | 38.43 | 38.43 | 38.43 | 43.11 | 43.11 |
| B21 | 11.632 | 10.394 | 11.263 | 11.691 | 11.775 | 8.489 | 6.278 | 6.699 | 8.986 |
| B22 | 0.020 | 0.019 | 0.018 | 0.018 | 0.016 | 0.009 | 0.008 | 0.014 | 0.014 |
| B23 | 0.039 | 0.039 | 0.039 | 0.040 | 0.040 | 0.040 | 0.040 | 0.039 | 0.038 |
| B24 | 0.014 | 0.013 | 0.012 | 0.012 | 0.011 | 0.007 | 0.007 | 0.010 | 0.010 |
| C11 | 15313.9 | 17427.7 | 18357.8 | 15657.4 | 16734.1 | 18061.2 | 19555.3 | 21108.1 | 22805.3 |
| C12 | 34.50 | 44.40 | 49.60 | 53.70 | 58.50 | 62.60 | 67.10 | 71.00 | 62.58 |
| C13 | 0.124 | 0.155 | 0.143 | 0.142 | 0.141 | 0.144 | 0.144 | 0.150 | 0.150 |
| C21 | 8.80 | 9.00 | 11.57 | 11.182 | 11.03 | 10.70 | 11.50 | 9.51 | 10.784 |
| C22 | 40.20 | 42.90 | 41.70 | 40.60 | 40.30 | 40.76 | 40.32 | 40.36 | 40.468 |
| C23 | 99.55 | 99.28 | 99.43 | 99.20 | 98.60 | 99.98 | 99.42 | 99.971 | 88.822 |
| D11 | 0.010 | 0.010 | 0.013 | 0.012 | 0.012 | 0.013 | 0.014 | 0.015 | 0.016 |
| D12 | 1.282 | 1.401 | 1.381 | 1.420 | 1.570 | 1.720 | 1.870 | 2.010 | 1.718 |
| D13 | 0.039 | 0.042 | 0.037 | 0.032 | 0.037 | 0.034 | 0.036 | 0.035 | 0.036 |
| D21 | 2.59 | 1.64 | 1.37 | 1.18 | 0.88 | 0.81 | 1.14 | 1.076 | 1.076 |
| D22 | 76.86 | 82.48 | 85.25 | 86.32 | 84.45 | 76.43 | 69.80 | 72.65 | 73.54 |
| **Sichuan** | | | | | | | | | |
|  | 2011 | 2012 | 2013 | 2014 | 2015 | 2016 | 2017 | 2018 | 2019 |
| A11 | 26133 | 29608 | 32617 | 35128 | 36775 | 40003 | 44651 | 48883 | 55774 |
| A12 | 0.093 | 0.084 | 0.075 | 0.072 | 0.067 | 0.064 | 0.060 | 0.060 | 0.057 |
| A21 | 14.20 | 13.812 | 13.00 | 12.40 | 12.236 | 11.931 | 11.526 | 10.88 | 10.30 |
| A22 | 38.613 | 39.552 | 38.85 | 39.593 | 40.632 | 41.787 | 42.721 | 43.605 | 43.926 |
| A31 | 52.50 | 51.663 | 51.70 | 48.90 | 44.082 | 40.835 | 38.745 | 37.67 | 37.30 |
| A41 | 33.40 | 34.526 | 35.20 | 38.70 | 43.682 | 47.234 | 49.729 | 51.40 | 52.40 |
| A42 | 32.00 | 32.80 | 33.40 | 34.10 | 34.80 | 35.60 | 36.20 | 36.90 | 35.52 |
| B11 | 2782.9 | 3587.2 | 3052.9 | 3148.5 | 2717.2 | 2843.3 | 2978.9 | 3548.2 | 3288.90 |
| B12 | 836.72 | 833.593 | 830.739 | 827.297 | 820.502 | 814.936 | 810.07 | 806.282 | 803.009 |
| B13 | 18.60 | 18.50 | 18.50 | 17.09 | 17.07 | 17.08 | 17.11 | 17.11 | 17.11 |
| B14 | 34.31 | 34.31 | 35.22 | 35.22 | 35.22 | 35.22 | 35.22 | 38.03 | 38.03 |
| B21 | 9.990 | 8.666 | 8.001 | 8.302 | 8.733 | 6.147 | 5.198 | 7.276 | 7.276 |
| B22 | 0.011 | 0.011 | 0.010 | 0.010 | 0.009 | 0.006 | 0.005 | 0.008 | 0.008 |
| B23 | 0.037 | 0.038 | 0.037 | 0.037 | 0.037 | 0.037 | 0.036 | 0.035 | 0.033 |
| B24 | 0.008 | 0.008 | 0.008 | 0.007 | 0.006 | 0.005 | 0.006 | 0.006 | 0.006 |
| C11 | 13559.5 | 15326.9 | 15513.2 | 14886.7 | 15957.9 | 17132.2 | 18499.9 | 19884.5 | 21483.6 |
| C12 | 26.30 | 48.30 | 52.60 | 56.50 | 59.60 | 62.80 | 67.90 | 71.80 | 63.72 |
| C13 | 0.146 | 0.182 | 0.167 | 0.156 | 0.167 | 0.163 | 0.160 | 0.151 | 0.153 |
| C21 | 12.60 | 13.34 | 14.59 | 14.223 | 13.519 | 12.90 | 14.46 | 13.18 | 13.656 |
| C22 | 38.20 | 38.70 | 38.40 | 37.51 | 38.65 | 39.90 | 40.00 | 40.55 | 39.322 |
| C23 | 88.43 | 88.29 | 94.98 | 95.40 | 96.79 | 98.60 | 98.54 | 99.296 | 99.817 |
| D11 | 0.010 | 0.011 | 0.011 | 0.012 | 0.013 | 0.013 | 0.012 | 0.015 | 0.018 |
| D12 | 1.399 | 1.470 | 1.516 | 1.575 | 1.670 | 1.720 | 1.720 | 1.810 | 1.699 |
| D13 | 0.025 | 0.025 | 0.026 | 0.025 | 0.023 | 0.021 | 0.023 | 0.023 | 0.026 |
| D21 | 0.670 | 0.750 | 0.890 | 1.010 | 0.720 | 0.880 | 0.830 | 0.866 | 0.866 |
| D22 | 47.10 | 45.89 | 41.27 | 43.42 | 44.32 | 38.46 | 39.20 | 41.334 | 41.334 |
| **Yunnan** | | | | | | | | | |
|  | 2011 | 2012 | 2013 | 2014 | 2015 | 2016 | 2017 | 2018 | 2019 |
| A11 | 19265 | 22195 | 25322 | 27264 | 28806 | 31093 | 34221 | 37136 | 47944 |
| A12 | 0.135 | 0.127 | 0.110 | 0.119 | 0.106 | 0.095 | 0.094 | 0.094 | 0.078 |
| A21 | 15.90 | 16.049 | 16.20 | 15.50 | 15.095 | 14.843 | 14.279 | 13.97 | 13.10 |
| A22 | 26.216 | 26.955 | 26.694 | 27.532 | 28.311 | 29.147 | 29.798 | 30.549 | 30.942 |
| A31 | 42.50 | 42.865 | 42.00 | 41.20 | 39.768 | 38.477 | 37.89 | 38.91 | 34.30 |
| A41 | 41.60 | 41.086 | 41.80 | 43.30 | 45.137 | 46.679 | 47.831 | 47.12 | 52.60 |
| A42 | 27.50 | 29.70 | 31.30 | 33.10 | 33.40 | 33.80 | 35.80 | 37.80 | 34.78 |
| B11 | 3206.50 | 3637.90 | 3652.20 | 3673.30 | 3959.31 | 4391.67 | 4602.41 | 4582.32 | 3166.40 |
| B12 | 1346.04 | 1336.10 | 1327.03 | 1316.80 | 1309.26 | 1301.15 | 1294.17 | 1286.40 | 1278.98 |
| B13 | 7.80 | 7.50 | 7.50 | 7.17 | 7.28 | 7.32 | 7.31 | 7.31 | 7.31 |
| B14 | 47.50 | 47.50 | 50.03 | 50.03 | 50.03 | 50.03 | 50.03 | 55.04 | 55.04 |
| B21 | 10.198 | 9.189 | 8.928 | 8.579 | 9.686 | 10.228 | 4.27 | 4.079 | 7.369 |
| B22 | 0.015 | 0.014 | 0.014 | 0.014 | 0.012 | 0.011 | 0.008 | 0.008 | 0.010 |
| B23 | 0.032 | 0.034 | 0.035 | 0.037 | 0.037 | 0.038 | 0.037 | 0.035 | 0.033 |
| B24 | 0.012 | 0.012 | 0.011 | 0.011 | 0.009 | 0.009 | 0.006 | 0.009 | 0.009 |
| C11 | 15533.1 | 17583.9 | 17974.7 | 16842.9 | 18131.2 | 19590.8 | 21133.7 | 22720.0 | 24335.3 |
| C12 | 43.30 | 41.80 | 44.80 | 47.70 | 50.10 | 53.10 | 57.20 | 60.30 | 53.68 |
| C13 | 0.165 | 0.189 | 0.167 | 0.152 | 0.163 | 0.174 | 0.175 | 0.177 | 0.158 |
| C21 | 10.06 | 10.25 | 11.61 | 12.363 | 12.619 | 13.17 | 13.60 | 12.97 | 12.944 |
| C22 | 38.70 | 39.30 | 37.80 | 38.14 | 37.27 | 37.84 | 38.87 | 39.78 | 38.38 |
| C23 | 74.13 | 82.70 | 87.62 | 92.50 | 90.03 | 92.96 | 92.74 | 98.162 | 99.772 |
| D11 | 0.010 | 0.009 | 0.010 | 0.010 | 0.010 | 0.009 | 0.009 | 0.009 | 0.009 |
| D12 | 0.631 | 0.667 | 0.675 | 0.671 | 0.800 | 0.890 | 0.950 | 1.050 | 0.872 |
| D13 | 0.033 | 0.028 | 0.026 | 0.025 | 0.028 | 0.030 | 0.031 | 0.028 | 0.030 |
| D21 | 1.34 | 1.28 | 1.68 | 1.19 | 1.03 | 0.99 | 0.86 | 1.15 | 1.15 |
| D22 | 49.00 | 49.50 | 52.46 | 49.83 | 51.00 | 49.82 | 38.46 | 38.90 | 45.602 |
| **Guizhou** | | | | | | | | | |
|  | 2011 | 2012 | 2013 | 2014 | 2015 | 2016 | 2017 | 2018 | 2019 |
| A11 | 16413 | 19710 | 23151 | 26437 | 29847 | 33246 | 37956 | 41244 | 46433 |
| A12 | 0.166 | 0.153 | 0.139 | 0.127 | 0.112 | 0.105 | 0.102 | 0.100 | 0.092 |
| A21 | 12.70 | 13.016 | 12.90 | 13.80 | 15.621 | 15.677 | 15.009 | 14.59 | 13.60 |
| A22 | 26.338 | 26.682 | 20.38 | 21.625 | 23.481 | 24.018 | 24.655 | 25.056 | 25.538 |
| A31 | 38.50 | 39.076 | 40.50 | 41.60 | 39.494 | 39.651 | 40.087 | 38.87 | 36.10 |
| A41 | 48.80 | 47.908 | 46.60 | 44.60 | 44.885 | 44.673 | 44.904 | 46.54 | 50.30 |
| A42 | 21.338 | 21.836 | 22.537 | 23.42 | 24.13 | 25.53 | 26.363 | 27.75 | 25.439 |
| B11 | 1802.1 | 2801.8 | 2174.2 | 3461.1 | 3278.7 | 3009.5 | 2947.4 | 2726.2 | 3092.90 |
| B12 | 1314.70 | 1306.60 | 1298.72 | 1294.21 | 1285.38 | 1274.32 | 1262.24 | 1255.22 | 1247.25 |
| B13 | 5.40 | 5.40 | 5.00 | 5.07 | 5.09 | 5.08 | 5.06 | 5.06 | 5.06 |
| B14 | 31.61 | 31.61 | 37.09 | 37.09 | 37.09 | 37.09 | 37.09 | 43.77 | 43.77 |
| B21 | 5.946 | 6.716 | 6.539 | 9.314 | 8.265 | 4.613 | 4.86 | 5.00 | 5.079 |
| B22 | 0.032 | 0.030 | 0.028 | 0.026 | 0.024 | 0.018 | 0.019 | 0.023 | 0.023 |
| B23 | 0.021 | 0.022 | 0.021 | 0.022 | 0.023 | 0.023 | 0.021 | 0.020 | 0.018 |
| B24 | 0.016 | 0.016 | 0.016 | 0.014 | 0.012 | 0.011 | 0.010 | 0.012 | 0.012 |
| C11 | 13453.5 | 15289.9 | 15515.3 | 15877.0 | 17192.8 | 18652.3 | 20210.7 | 21875.8 | 23647.9 |
| C12 | 29.70 | 40.00 | 47.60 | 51.90 | 55.70 | 59.20 | 65.10 | 68.20 | 60.02 |
| C13 | 0.168 | 0.182 | 0.182 | 0.180 | 0.196 | 0.198 | 0.196 | 0.196 | 0.179 |
| C21 | 8.70 | 8.80 | 9.60 | 10.615 | 11.267 | 11.36 | 11.02 | 10.96 | 11.044 |
| C22 | 32.30 | 32.80 | 34.50 | 33.97 | 35.88 | 36.80 | 37.01 | 38.64 | 36.46 |
| C23 | 88.56 | 91.92 | 92.23 | 93.30 | 93.81 | 94.65 | 95.22 | 96.092 | 96.593 |
| D11 | 0.009 | 0.011 | 0.011 | 0.013 | 0.015 | 0.016 | 0.019 | 0.020 | 0.019 |
| D12 | 0.637 | 0.609 | 0.584 | 0.600 | 0.590 | 0.630 | 0.710 | 0.820 | 0.670 |
| D13 | 0.025 | 0.024 | 0.026 | 0.024 | 0.024 | 0.027 | 0.027 | 0.027 | 0.032 |
| D21 | 1.140 | 1.010 | 1.370 | 1.840 | 1.310 | 1.010 | 1.600 | 1.426 | 1.426 |
| D22 | 52.70 | 61.76 | 50.77 | 58.33 | 59.80 | 58.10 | 54.70 | 64.60 | 63.60 |

**The initial values of variables in the fsQCA model**

|  | [Province](C:/Users/ncdx/AppData/Local/Youdao/dict/Application/8.9.6.0/resultui/html/index.html#/javascript:;)  [and](C:/Users/ncdx/AppData/Local/Youdao/dict/Application/8.9.6.0/resultui/html/index.html#/javascript:;) [city](C:/Users/ncdx/AppData/Local/Youdao/dict/Application/8.9.6.0/resultui/html/index.html#/javascript:;) | EDL | STIL | IS | OL | ER | US | GDL |
| --- | --- | --- | --- | --- | --- | --- | --- | --- |
| 2011 | Shanghai | 82560 | 47960 | 41.3 | 3774 | 96.56 | 89.3 | 0.1450 |
|  | Jiangsu | 62290 | 199814 | 51.3 | 5729 | 94.89 | 61.9 | 0.1120 |
|  | Zhejiang | 59249 | 130190 | 51.2 | 2019 | 91.07 | 62.3 | 0.1180 |
|  | Anhui | 25659 | 32681 | 54.3 | 329 | 78.7 | 44.8 | 0.0815 |
|  | Jiangxi | 26150 | 5550 | 54.6 | 491 | 55.27 | 45.7 | 0.0756 |
|  | Hubei | 34197 | 19035 | 50 | 519 | 79.08 | 51.83 | 0.0766 |
|  | Hunan | 29880 | 16064 | 47.6 | 350 | 66.2 | 45.1 | 0.0820 |
|  | Chongqing | 34500 | 15525 | 55.4 | 452 | 76.86 | 55.02 | 0.0849 |
|  | Sichuan | 26133 | 28446 | 52.5 | 574 | 47.1 | 41.83 | 0.0754 |
|  | Yunnan | 19265 | 4199 | 42.5 | 206 | 49 | 36.8 | 0.0809 |
|  | Guizhou | 16413 | 3386 | 38.5 | 57 | 52.7 | 34.96 | 0.0681 |
| 2015 | Shanghai | 103795.5 | 60623 | 31.807 | 6612.74 | 96.15 | 87.6 | 0.1371 |
|  | Jiangsu | 87995 | 250290 | 45.702 | 7821.54 | 95.28 | 66.52 | 0.1096 |
|  | Zhejiang | 77643.69 | 234983 | 45.962 | 2918.13 | 92.55 | 65.8 | 0.1063 |
|  | Anhui | 35996.56 | 59039 | 49.746 | 1064.87 | 88.48 | 50.5 | 0.0811 |
|  | Jiangxi | 36724 | 24161 | 50.297 | 725.782 | 57.03 | 51.62 | 0.0739 |
|  | Hubei | 50653.85 | 38781 | 45.697 | 892.313 | 67.78 | 56.85 | 0.0762 |
|  | Hunan | 42753.86 | 34075 | 44.325 | 521.472 | 65.4 | 50.89 | 0.0967 |
|  | Chongqing | 52321 | 38914 | 44.978 | 788.446 | 84.45 | 60.94 | 0.0895 |
|  | Sichuan | 36775 | 64953 | 44.082 | 884.094 | 44.32 | 47.69 | 0.0879 |
|  | Yunnan | 28806 | 11658 | 39.768 | 327.196 | 51 | 43.33 | 0.0716 |
|  | Guizhou | 29847.248 | 14115 | 39.494 | 181.469 | 59.8 | 42.01 | 0.07 |
| 2019 | Shanghai | 157279 | 100587 | 27 | 7218.34 | 95.278 | 88.3 | 0.1394 |
|  | Jiangsu | 123607 | 314395 | 44.4 | 8803.97 | 94.158 | 70.61 | 0.1077 |
|  | Zhejiang | 107624 | 285342 | 42.6 | 3387.57 | 93.8 | 70 | 0.0939 |
|  | Anhui | 58495.6 | 82524 | 41.3 | 842.735 | 87.372 | 55.81 | 0.0983 |
|  | Jiangxi | 53164 | 59140 | 44.2 | 771.666 | 46.368 | 57.42 | 0.0778 |
|  | Hubei | 77386.5 | 73940 | 41.7 | 1047.25 | 66.162 | 61 | 0.0848 |
|  | Hunan | 57540.3 | 54685 | 37.6 | 1006.05 | 70.301 | 57.22 | 0.0899 |
|  | Chongqing | 75828 | 43872 | 40.2 | 879.307 | 73.54 | 66.8 | 0.0825 |
|  | Sichuan | 55774 | 82066 | 37.3 | 1007.51 | 41.334 | 53.79 | 0.0848 |
|  | Yunnan | 47944 | 22324 | 34.3 | 365.551 | 45.602 | 48.91 | 0.0736 |
|  | Guizhou | 46433 | 24729 | 36.1 | 267.84 | 63.6 | 49.02 | 0.0672 |
